# Spatial Patterns and Epidemiological Drivers of Foot‐and‐Mouth Disease Outbreaks in Uganda

**DOI:** 10.1155/tbed/4994209

**Published:** 2026-07-21

**Authors:** Lina González Gordon, Dennis Muhanguzi, Adrian Muwonge, Sylvester Ochwo, Noelina Nantima, Rose Ademun, Lisa Boden, Norbert Frank Mwiine, Barend Mark de C. Bronsvoort, Thibaud Porphyre

**Affiliations:** ^1^ Division of Epidemiology, The Roslin Institute, Royal (Dick) School of Veterinary Studies, University of Edinburgh, Midlothian, UK, ed.ac.uk; ^2^ Department of Biomolecular Resources and Biolaboratory Sciences, College of Veterinary Medicine, Makerere University, Kampala, Uganda, mak.ac.ug; ^3^ Department of Animal Health, Ministry of Agriculture Animal Industry and Fisheries, Entebbe, Uganda; ^4^ Center for Animal Health and Food Safety, College of Veterinary Medicine, University of Minnesota, Saint Paul, USA, umn.edu; ^5^ Division of Global Agriculture and Food Systems, Royal (Dick) School of Veterinary Studies, University of Edinburgh, Midlothian, UK, ed.ac.uk; ^6^ Laboratoire de Biométrie et Biologie Évolutive, Université Claude Bernard Lyon 1, CNRS, VetAgro Sup, Marcy l’Étoile, France, univ-lyon1.fr

**Keywords:** Bayesian disease modeling, cattle, disease outbreaks, Eastern Africa, foot-and-mouth disease, geographic mapping, INLA, risk estimation, spatial analysis, vaccination

## Abstract

Foot‐and‐mouth disease (FMD) places a heavy economic burden on farmers and animal health authorities due to its clinical effects, the high cost associated with its prevention and control, and trade restrictions on livestock and livestock products in countries without FMD‐free recognition like Uganda. Building on our previous work using cattle movement networks for risk mapping, we demonstrate that Bayesian disease mapping can serve as a complementary epidemiological approach to identify and communicate spatial variation in outbreak risk. A Bayesian Poisson mixed‐effects spatial regression model was built with the Integrated Nested Laplace Approximation (INLA) to analyze FMD outbreak data from 2014 to 2019. This analysis examined the spatial variation in risk and assessed potential drivers of its distribution, providing insights to support targeted and cost‐effective disease surveillance and control strategies. Cattle density, Enhanced Vegetation Index (EVI), deprivation score, and human density were associated with the risk of outbreaks but did not explain most of its spatial distribution, suggesting that additional epidemiological factors may contribute and should be examined in future models. Mean vaccination coverage was associated with an increased risk of outbreaks, consistent with the predominantly reactive vaccination program implemented during the study period. Adjusted district‐specific risk estimates and exceedance probabilities (EPs) highlighted districts across the cattle corridor, a wide zone that extends from the southwestern to the northeastern part of the country, as areas of greater risk. Lower‐risk areas mapped to large parts of the northern and western regions, a finding that may reflect limited disease detection, given the past evidence of viral exposure in these areas. A better understanding of district‐specific factors contributing to the outbreaks, together with a timely and sensitive surveillance system, is crucial for supporting evidence‐based FMD control plans. Such strategies should account for the characteristics of local communities, the typical operation of livestock production systems, and their unique challenges to improve their effectiveness.

## 1. Introduction

Foot‐and‐mouth disease (FMD), caused by the FMD virus (FMDV; genus *Aphthovirus*, family *Picornaviridae*), is a highly contagious, transboundary disease circulating within wild and domestic cloven–hoofed animals. Despite its low case fatality among adult animals, the disease is associated with high morbidity and is often fatal in young animals [1]. In susceptible livestock populations, FMDV triggers large‐scale epidemic outbreaks that significantly compromise animal welfare, reduce productivity, and decrease the profitability of the agricultural sector, threatening local livelihoods and food security [2–5]. As a result, FMD is one of the most important livestock diseases and has been targeted for elimination with an official global control strategy launched in 2012 [6, 7].

In East Africa, extensive viral genetic diversity, absence of serotype cross‐protective immunity, and multihost interactions across susceptible domestic and wildlife species facilitate continuous viral introductions and rapid local spread via multiple direct and indirect pathways [1, 8–12]. This complex epidemiological landscape is reflected in the recurrence of outbreaks linked to immunologically diverse serotypes within virus pool 4 (O, A, SAT‐1, SAT‐2, and SAT‐3) [1, 13]. Suppressive vaccination, livestock‐movement standstills, and market closures have not resulted in sustained changes in disease circulation, and outbreaks continue to periodically occur across the region [8–10, 14, 15]. Although the intricacies of viral shifts are best studied through phylodynamic analysis and sequence modeling, outbreak data provide valuable insights into how eco‐epidemiological processes and externalities, such as vaccination, influence epidemic waves involving multiple host species [8–10, 15, 16]. At broader spatial scales, transboundary and within‐country movements, geographical accessibility, the distribution of susceptible species, animal‐gathering points, trade networks, and socioeconomic, political, and environmental factors can all influence viral turnover, transmission, and persistence [11, 12, 15, 16]. These interconnected aspects shape the biogeography of FMD and should, wherever possible, be incorporated into statistical and mathematical models designed to map and project FMD risk in endemic settings [9, 15, 17]. This integrated approach contrasts with the typically narrow scope of many disease models, which tend to focus on a single process or perspective (e.g., social or environmental), thereby lacking a systems‐level view of disease risk [18, 19].

In Uganda, serological, outbreak, and sequence data have been used to assess FMD risk over the years. Phylodynamic and phylogeographic models have explored the origin, relatedness, trajectory, and dispersal patterns of FMDV across the country [11, 12, 20–22], while serological surveys have quantified viral exposure in both naïve and vaccinated populations of wildlife ungulates, cattle, small ruminants, and pigs [22–24]. These studies have identified several individual and population‐level risk factors; however, the evidence remains fragmented as most data lack country‐wide coverage. Similarly, the relationship between local epidemiological factors and outbreak risk remains poorly understood, partly because of challenges in collecting, harmonizing, and analyzing historical outbreak data within a dynamic administrative landscape marked by decentralization and the creation of new districts [15, 25–28] and partly due to reporting inconsistencies that make the interpretation of outbreak data challenging [26]. In spite of its limitations, routine outbreak data collected by animal health services in Uganda offer a unique opportunity to model disease risk, identify hotspots, and evaluate the association between local epidemiological factors and variations in spatial risk patterns [29–32].

Practical approaches for geographic prioritization and targeted resource allocation can help optimize the design of disease control plans, particularly in resource‐constrained settings [33, 34]. Disease‐mapping models provide a powerful framework for examining spatial heterogeneity in disease occurrence and identifying localized processes shaping its distribution [35, 36]. Modeling approaches that use outbreak data for spatial risk estimation in FMD‐endemic settings often fail to adequately account for spatial dependencies, uncertainty, and the full range of epidemiological factors shaping disease patterns within imperfect disease detection systems [15]. Incorporating these aspects is therefore crucial for strengthening inferences from spatial models in complex epidemiological environments and improving the interpretability of the resulting spatial risk patterns.

Here, we aim to identify which epidemiological factors linked to district‐level accessibility, connectivity, animal demographics, trade, socioeconomics, and the environment (understood as a combination of climate and natural resources) influence spatial disease patterns, outbreak risk, and disease detection at a broader spatial scale [15, 37]. To do so, we analyzed FMD spatial patterns across Uganda by modeling the district‐level number of outbreaks reported between 2014 and 2019 and examined the effects of epidemiological factors and externalities, such as vaccination coverage, on district‐level outbreak risk. In addition, we investigated the robustness of posterior estimates and spatial risk patterns under alternative assumptions about spatial variation. All analyses were conducted within a Bayesian hierarchical framework for disease mapping. By leveraging routine passive surveillance data, this analysis aims to generate context‐specific insights on localized disease risk and its drivers to enhance animal health intelligence, epidemic preparedness, and better guide resource allocation for cost‐effective disease control.

## 2. Materials and Methods

### 2.1. Study Area and Outbreak Response Context

Uganda is a land‐locked East African country with a geographical extension of ~241,500 km^2^. The country is divided into four major regions and 15 subregions (Supporting Information: File 1). Districts are the highest administrative units, operating with devolved administrations where government‐led veterinary services are responsible for the surveillance, prevention, and control of FMD and other WOAH‐listed diseases. Suspected FMD cases are reported by farmers to community animal health workers (AHWs) and veterinarians, triggering local outbreak investigations coordinated by the district veterinary officer (DVO). Samples are collected from affected premises for confirmation by Ag‐ELISA or PCR. Diagnostic support, including the provision of sampling kits, reagents, and sequencing services, is provided through the WOAH/FAO FMD Reference Laboratory Network, coordinated locally by the National Animal Disease Diagnostics and Epidemiology Center (NADDEC) in collaboration with the National Agricultural Research Organization (NARO). District‐level movement bans and market closures are imposed during outbreaks, followed by emergency vaccination in affected areas. Outbreak response measures are implemented for variable periods and over a geographical scale determined for each outbreak, remaining in place until the outbreak is contained and/or vaccination in at‐risk areas is completed.

### 2.2. Empirical Outbreak and Vaccination Data

The Ministry of Agriculture, Animal Industry and Fisheries (MAAIF) provided the outbreak and vaccination data used in this analysis. The outbreak dataset, spanning January 2014 to December 2019, was compiled from monthly records from DVOs and includes the outbreak date, location, and species affected. In Uganda, the Risk‐Based Strategic Plan for FMD control defines an outbreak as the occurrence of one or more laboratory‐confirmed FMD cases within a specific epidemiological unit, such as a farm, parish, or subcounty [38]. To ensure consistency in spatial resolution across the study period, the geographical accuracy of each outbreak, usually reported at the village level, was verified to the lowest possible administrative level for each year and standardized to align with the 2019 district boundaries. Official FMD vaccination records covered the period from January 2015 to December 2019 and consisted of the total number of FMD vaccine doses assigned in each district. Uganda typically stocks inactivated multivalent FMD vaccines, either trivalent (with serotypes A, O, and SAT‐1) or tetravalent (with serotypes SAT‐1, SAT‐2, O, and A) [39]. In accordance with national FMD control policies in place in Uganda during the period covered by this analysis, vaccination data are most likely representative of reactive vaccination efforts rather than routine, mass vaccination campaigns. The unit of analysis for this model was the district, with the outcome defined as the total number of FMD outbreaks aggregated at the district level for the whole study period.

### 2.3. Epidemiological Variables and Data Sources

Epidemiological factors considered in relation to FMD outbreak risk were grouped into five themes: (a) animal demographics and interactions, (b) spatial accessibility, (c) trade, (d) socioeconomic, and (e) environmental based on a classification scheme proposed by the authors (Supporting Information: Files 2, 3) [15]. All variables were compiled at the district level for a fixed period within the observation window of the outcome. Because Uganda has two mostly distinct dry seasons (December–February and June–August) and two wet periods (March–May, short rains, and September–November, long rains) [40], the Enhanced Vegetation Index (EVI) was the only predictor incorporating spatiotemporal structure. The 16‐day vegetation index at 1 km (M ^∗^D13A3) was used to generate an EVI raster time series from MODIS Land Products data, processed with the *MODIStsp* package to produce seasonal district‐level means for the dry and wet periods [41, 42]. District‐level livestock population density was calculated using the FAO Gridded Livestock of the World database (GLW v3.1, 2015) [43]. Human population projections per district, publicly available at the Ugandan Bureau of Statistics (UBOS), were used to calculate human density at the district level [44]. Statistics on urban population, standard of living, and economic poverty were compiled from local organizations working in global health and socioeconomic development [45]. Estimates of subnational GDP per capita were obtained from remote sensing estimations [46]. Road density and water availability were derived from official national shapefiles. District centrality measures from previous work, computed from the annual network of between‐district cattle movements in 2019, were used to represent within‐country trade [47]. Transboundary agropastoral movements and trade were captured by classifying districts as either located at the border with neighboring countries or not. District‐level annual cattle vaccination coverage was calculated using the “distribution method” as the number of vaccine doses distributed divided by the estimated cattle population size [48]. Mean vaccination coverage for the period 2015–2024 was then computed for each district using values across all years.

### 2.4. Spatial Analysis

#### 2.4.1. Modeling Framework

Bayesian hierarchical models (BHMs) were used to analyze reported district‐aggregated outbreak counts. This type of model incorporates spatial dependence and ecological regression for multivariable disease mapping [32, 36, 49–52]. Models were implemented using the Integrated Nested Laplace Approximation (INLA) via the *INLA* package (Version 25.10.19) [49, 50]. All analyses were conducted in R Version 4.5.1, using *tidyverse*, *sp*, and *spdep* packages for data processing and visualization [53–55].

#### 2.4.2. Model Structure

The number of outbreaks *y*_*i*_ was modeled for each district *i* in terms of the observed‐to‐expected ratio (*θ*_*i*_) or standardized morbidity ratio (SMR), where *e*_*i*_ is the expected number of outbreaks and is calculated by standardizing outbreak counts to the number of villages at risk per district (Supporting Information: File 4). We assumed *y*_*i*_ follow a Poisson distribution such as:

yi ∼Poisson eiθi,

logθi=bo+∑q=1kbqxqi+ui+vi+zi,

where *b*_*o*_ is the intercept and quantifies the adjusted average district‐level outbreak risk over the whole country; *b*_*q*_ represents the coefficients for each *q* explanatory variable *x*_*q*_, with *q* ∈ {1, …, *k*}; (*u*_*i*_ +  *v*_ *i*_) denotes the unstructured and structured spatial random effect for each of the 135 districts [36, 51]; and *z*_*i*_ is an independent and identically distributed random effect accounting for correlations due to districts being in the same subregion (*n* = 15).

Here, the spatial random effect (*u*_*i*_ + *v*_*i*_) links each observation to its area, accounting for district‐ or subregion‐specific effects not explained by the covariables. The Besag‐York‐Molliè (BYM) convolution model [56], commonly used for disease mapping, was the spatial structure selected for this model [32, 35, 36, 49]. Further details on these three components and underlying calculations are provided in Supporting Information: File 4.

Default prior distributions set in the INLA package were used for the fixed effects (~*N*(0, 0.001)) and intercept (~*N*(0, 0)) with noninformative (vague) default hyperpriors used for spatial modeling (~LogGamma(1, 0.0005)) [50].

#### 2.4.3. Variable Selection

A staged, sequential approach was used to select the subset of covariates that best explained outbreak counts. Variables were first screened in univariable analyses, and those for which the posterior distribution did not overlap zero within an 80% credible interval (CrI) were retained to construct a global model. To avoid issues of model fit and interpretation, explanatory variables within each risk class were assessed for multicollinearity using Kendall’s rank correlation and the variance inflation factor (VIF) and only one retained (VIF > 5 or Kendall > 0.6) [57, 58]. Finally, a set of submodels was generated for comparison using a stepwise backward elimination approach [36]. Alternative model formulations were compared to identify the model that best described the data, balancing the magnitude of the posterior estimates with their biological and epidemiological importance [59]. Model comparison was based on the Deviance Information Criteria (DIC), the Watanabe‐Akaike Information Criteria (WAIC), and marginal log likelihoods to achieve the best possible trade‐off between model fit, complexity, and appropriateness given the data [36, 51, 60–62].

#### 2.4.4. Goodness‐of‐Fit

Several methods were used to assess the adequacy of the best candidate model [62]. Cross‐validation through posterior predictive checks was used to assess the model fit based on its predictive distribution; the posterior predictive distribution was plotted against the observed outbreak counts to represent the likelihood of a replicate observation having observed the data [35, 51]. Moreover, deviance‐based methods were used as indications of local model performance, with lower local DIC values indicating a better fit [36]. Calculating goodness‐of‐fit measures such as local DIC allows to incorporate the uncertainty on the assessment of how well the selected model captures risk within a particular area in light of the local spatial structure and covariate patterns, improving the interpretability of the model outputs [63].

#### 2.4.5. Local Risk Estimation and Identification of High‐Risk Areas

Exceedance probabilities (EPs) were calculated to indicate how often the district‐level relative risk (RR) of the spatial random effects (RR = exp (*u*_*i*_ + *v*_*i*_)) exceeded the null risk value. These probabilities were used to inform local disease risk and to identify localized spatial behavior, highlighting areas of unusually high (“hotspots”) or low outbreak risk. EPs were calculated as $\;q_{i}^{c}=\mathrm{Pr}\left(u_{i}+v_{i}>c\right)$, where *c* = 0. Different threshold levels for the RR can be evaluated as previously suggested for studies analyzing small‐area health data, including FMD [31, 64, 65].

#### 2.4.6. Alternative Data Distributions and Sensitivity Analysis

In addition to the Poisson model, alternative data count models were assessed at the early stages of selecting the best model structure: the negative binomial (NB) model, to address overdispersion potentially arising from excess variation in outbreak counts between districts, and the zero‐inflated Poisson (ZIP) and zero‐inflated NB (ZINB) models, to address the possibility of excess zeros in the data [66, 67]. In Uganda, districts without outbreak records for the studied period may represent a true absence of outbreaks (structural zeros), undetected or unreported outbreaks (nonstructural zeros), or a combination of both [26, 28, 68]. Because the available data do not allow for the source of zeros to be clearly distinguished, uncertainty remains regarding the mechanisms generating zero observations in the dataset, supporting the inclusion of zero‐inflated models as part of the analytical pipeline [66]. Zero inflation was implemented using the Type I option in the INLA model specification (structural and nonstructural zeros). All these model structures were compared based on the DIC, WAIC, and marginal log likelihoods for a model that included only the random effects and assessed across selected probability distributions. The two best model structures were moved forward for model comparison and specified with explanatory covariates only, as well as models incorporating covariates plus alternative spatial random‐effect structures at the district level or at both the district and subregion level. A sensitivity analysis using three hyperpriors representing small, intermediate, and extreme geographical variation, as proposed by Berardinelli et al. [69], was used to check the robustness of the posterior estimates of the best model. These priors are widely applicable to small‐area health modeling [61, 70]. Lastly, vaccination coverage was first analyzed independently in relation to outbreak counts and subsequently incorporated into the best‐fitting spatial model after adjustment for key epidemiological variables. Results are therefore presented for the best‐fitting models with and without vaccination coverage.

## 3. Results

### 3.1. Spatial Distribution of Outbreaks

**Figure 1:**
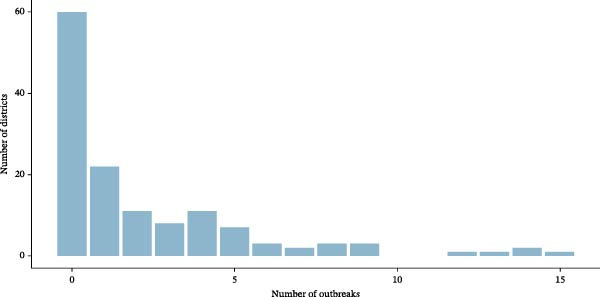
Breakdown of total outbreak reports at the district level, 2014–2019. Most districts reported no outbreaks during the study period (*n* = 60), while the maximum number of reported outbreaks in a single district reached 15.

**Figure 2:**
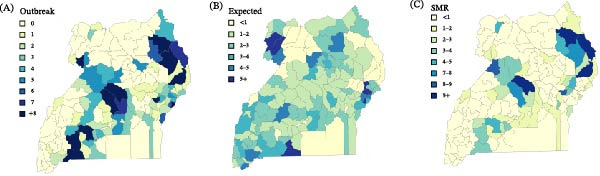
Spatial patterns of data used for Bayesian modeling across Ugandan districts. (A) Reported FMD outbreaks from 2014 to 2019. (B) Expected outbreaks calculated by standardizing outbreak counts to the number of villages at risk in each district. (C) Baseline standardized morbidity ratio (SMR), representing the ratio of observed to expected outbreak counts.

Between 2014 and 2019, a total of 298 FMD outbreaks were reported in cattle across all districts (median: 1 and IQR: 3.5). Sixty districts (44.4%) did not report outbreaks during the study period (Figure 1). Baseline risk estimates showed that 64.4% of the districts (*n* = 87) were below their calculated outbreak expectation (SMR < 1); these districts extend across the north and southeast of the country, geographically mapping to Acholi, West Nile, Lango, Toro, and Kigezi subregions. By comparison, 35.6% (*n* = 48) of the districts presented SMR > 1, meaning a higher baseline outbreak risk (between 1.01 and 18.4), and these were predominantly distributed along the cattle corridor, a zone spanning from the southwest to the northeast of the country, characterized by pastoral rangelands and a diversity of livestock systems. The largest number of outbreaks was observed along Karamoja (17.8%), Buganda (North: 16.8% and South: 11.1%), Elgon (11.7%), and Ankole (10.7%), with significant differences in subregional outbreak reports (Kruskal–Wallis, *p* < 0.05; Supporting Information: File 1). The contrast between the raw and expected outbreak counts and the SMRs for each district is shown in Figure 2.

### 3.2. Model Structure and Variable Selection

Four probability distributions (Poisson, ZIP, NB, and ZINB) were initially explored with the spatial random effects only. Among the candidate baseline spatial models, the Poisson model showed the lowest values of DIC and WAIC, suggesting that the spatial random effects sufficiently captured overdispersion and zero inflation in the outbreak counts (Supporting Information: File 5). All population‐level covariates were individually screened as the initial set of candidate predictors (Supporting Information: File 6). To address collinearity and avoid including highly correlated variables, only a limited number of variables from each conceptual risk domain were taken forward into multivariable modeling. Covariates were retained if they demonstrated evidence of association with the outcome or if their exclusion increased the DIC, thereby supporting a parsimonious model that appropriately adjusts for confounding (Supporting Information: File 7).

### 3.3. Best Model and Ecological Regression Results

The results of all the Poisson models compared are shown in Table 1. All models included the same explanatory variables (cattle density, degree centrality, human density, deprivation, poverty, and EVI during the second dry season), which were selected through univariable screening but differed in the structure of the spatial effects. The model including both structured and unstructured random effects at the district level (Model 2; BHM spatial Poisson—district) provided the best balance between model fit and parsimony (DIC = 417.66; WAIC = 432.57) and was therefore selected as the best model. For comparison, the model including both district and subregion random effects (Model 3; BHM spatial Poisson—district and subregion) performed similarly in terms of model fit but added complexity due to the inclusion of the subregion (DIC = 417.12; WAIC = 431.41). Model 1 (covariates only, without a spatial term) performed poorly across all model selection metrics and showed strong residual spatial autocorrelation (Moran’s *I*, *p* < 0.01), further supporting the need to account for the spatial structure.

**Table 1:** Results of Bayesian Poisson ecological regression models with different spatial structures.

Variable	Summary: mean ± SD	Model 1: Poisson—no spatial terms RR (95% CrI)	Model 2: BHM spatial Poisson—district RR (95% CrI)	Model 3: BHM spatial Poisson—district and subregion RR (95% CrI)
Cattle density	66.22 ± 50.60	3.90 (2.29–6.62)	8.00 (2.39–28.79)	8.08 (2.39–29.08)
Network degree category				
Q5	40.90 ± 14.10	Ref.		
Q4	18.20 ± 3.29	0.55 (0.36–0.85)	0.83 (0.33–2.10)	0.83 (0.33–2.12)
Q3	9.96 ± 1.89	0.84 (0.58–1.19)	1.77 (0.79–4.10)	1.79 (0.79–4.10)
Q2	5.22 ± 1.01	0.39 (0.24–0.63)	0.75 (0.28–2.01)	0.75 (0.28–2.03)
Q1	2.30 ± 1.38	0.73 (0.51–1.06)	1.23 (0.44–3.53)	1.23 (0.43–3.56)
Human density	301.06 ± 732.69	0.21 (0.14–0.31)	0.18 (0.05–0.64)	0.18 (0.05–0.64)
EVI 2nd dry season category				
Q5	0.47 ± 0.01	Ref.		
Q4	0.43 ± 0.01	0.84 (0.54–1.30)	1.05 (0.38–2.92)	1.05 (0.37–2.94)
Q3	0.40 ± 0.01	1.20 (0.78–1.82)	2.20 (0.75–6.62)	2.20 (0.75–6.69)
Q2	0.37 ± 0.01	0.99 (0.65–1.51)	2.97 (1.01–9.12)	3.00 (1.00–9.21)
Q1	0.28 ± 0.09	0.89 (0.57–1.38)	1.99 (0.64–6.49)	2.01 (0.64–6.55)
Deprivation score category				
Q5	47.62 ± 7.48	Ref.		
Q3	35.95 ± 1.22	0.63 (0.43–0.90)	1.09 (0.43–2.89)	1.11 (0.43–2.89)
Q3	29.48 ± 2.69	0.53 (0.35–0.79)	0.58 (0.21–1.55)	0.58 (0.21–1.57)
Q2	22.57 ± 2.05	1.04 (0.70–1.55)	0.84 (0.31–2.25)	0.84 (0.31–2.27)
Q1	13.94 ± 3.68	0.24 (0.13–0.43)	0.20 (0.05–0.70)	0.20 (0.05–0.71)
Poverty category				
Q5	49.41 ± 10.42	Ref.		
Q4	32.37 ± 2.88	0.51 (0.33–0.77)	0.69 (0.21–2.25)	0.69 (0.21–2.27)
Q3	20.95 ± 3.05	0.58 (0.38–0.90)	1.72 (0.49–6.17)	1.72 (0.49–6.23)
Q2	15.58 ± 0.90	0.70 (0.45–1.07)	1.31 (0.34–5.26)	1.32 (0.33–5.31)
Q1	10.31 ± 3.10	0.54 (0.35–0.84)	2.01 (0.44–9.58)	2.03 (0.44–9.68)
Model fit				
DIC	—	665.75	417.66	417.12
WAIC	—	727.75	432.57	431.41

*Note:* Q1–Q5 represent quintile groupings.

In the best‐fitting model (BHM spatial Poisson—district), cattle density, human population density, deprivation score, and EVI were associated with the spatial distribution of risk. Higher cattle density was associated with an increased risk of outbreaks (RR = 8.00; 95% CrI: 2.39–28.79). Districts with lower mean EVI during the second dry season showed increased outbreak risk (Q2 vs. Q5: RR = 2.97; 95% CrI: 1.01–9.12), though the lowest EVI category (Q1) was not associated (RR = 1.99; 95% CrI: 0.64–6.49). Districts with the lowest standard of living (deprivation score Q1: RR = 0.20; 95% CrI: 0.05–0.70) and higher human population density were at lower risk (RR = 0.18; 95% CrI: 0.05–0.64).

### 3.4. Outbreak Spatial Risk and Disease Cluster Detection

The model‐based estimates and baseline risk (Figure 2C) showed a similar geographical distribution when mapped as SMRs (Supporting Information: File 8). However, the fitted risk map represents smoothed outbreak estimates after adjustment for epidemiologically relevant covariates and spatial variation through the inclusion of spatial autocorrelation and unstructured heterogeneity.

**Figure 3:**
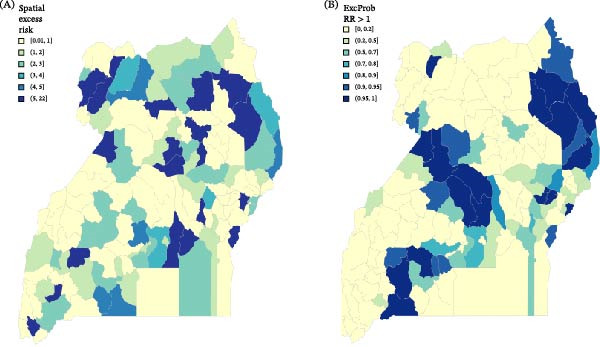
District‐level FMD risk across the country based on the best‐fitting model for 2014–2019. (A) Spatial excess relative risk (RR), indicating residual spatial variation not fully explained by the covariates included in the model. (B) Exceedance probabilities (RR > 1); darker blue indicates a higher probability (high‐risk clusters).

Spatial excess risk derived from the district‐level spatial random‐effect residuals (Figure 3A) indicated that several districts in the north, northwest, and southwest of the country, where outbreak reports were relatively rare (Figure 2A), exhibited elevated residual spatial risk, suggesting the presence of underlying epidemiological aspects not fully captured by the covariates included in the model. Posterior variance decomposition suggested strong spatial autocorrelation (~99%) and comparatively little district‐specific heterogeneity beyond the spatial pattern. Posterior EPs (Figure 3B), interpreted as the probability that a district has a RR > 1, identified high‐risk clusters (posterior probability > 90%) in the Karamoja, Bunyoro, Buganda, and Ankole subregions. In contrast, low‐risk clusters (posterior probability < 20%) encompassed much of the West Nile, Acholi, Lango, Teso, Toro, and Kigezi subregions. Districts with the greatest uncertainty (posterior probability ~50%) were predominantly located around high‐risk areas, particularly in Lango, Bunyoro, and North and South Buganda.

### 3.5. Vaccination Coverage

**Figure 4:**
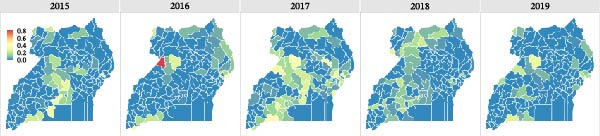
Annual cattle FMD vaccination coverage between 2015 and 2019. Maps depict the mean district‐level vaccination coverage, calculated as the total number of vaccine doses assigned per year divided by the cattle population of each district, illustrating the heterogeneous spatial distribution of vaccination coverage.

For the period 2015–2019, the spatial distribution and mean district‐level vaccination coverage were heterogeneous (Figure 4). The distribution of cattle vaccines covered a broader geographic area in 2017, when 68 districts (50.4% of the country) received vaccine doses, corresponding to a mean district‐level vaccination coverage of 14.78% (95% CI: 12.73–17.13) among districts where vaccines were distributed. In contrast, vaccines were distributed in 31 districts in 2015 (23.0%; mean district‐level coverage among vaccinated: 13.69%, 95% CI: 9.86–17.52), 29 districts in 2016 (mean district‐level coverage among vaccinated: 21.5%; 14.14%, 95% CI: 7.91–20.38), 47 districts in 2018 (mean district‐level coverage among vaccinated: 34.8%; 10.40%, 95% CI: 8.50–12.31), and 22 districts in 2019 (16.3%; mean district‐level coverage among vaccinated: 13.00%, 95% CI: 9.71–16.29). A total of 40 districts (29.5%) did not receive any FMD vaccine doses between 2015 and 2019. Among the districts that received vaccines at least once during this period, the mean 5‐year vaccination coverage was the highest in Isingiro (25.39%; range: 10.98–44.18), Kiryandongo (24.13; range: 9.07–36.29), Buliisa (19.97%; range: 0.00–83.20), Kapchorwa (16.81%; range: 0.00–28.02), and Koboko (15.48%; range: 8.06–35.17).

Higher mean vaccination coverage for the period 2015–2019 was associated with an increased FMD risk (RR = 1.15; 95% CrI: 1.08–1.22), consistent with a predominantly reactive vaccination program targeted to historically high‐risk districts (BHM spatial Poisson—district + vaccination; ΔDIC = 3.38; ΔWAIC = 6.44). Including mean vaccination coverage as a variable in the best‐fitting model did not alter the direction or substantially change the magnitude of the associations for cattle (RR = 8.17; 95% CrI: 2.61–26.58) and human density (RR = 0.17; 95% CrI: 0.06–0.51). However, deprivation was no longer associated with outbreak risk, and the association between EVI in the 2nd dry season and outbreak risk became more prominent (Q2 vs. Q5: RR = 3.86; 95% CrI: 1.45–10.70 & Q3 vs. Q5: RR = 2.72; 95% CrI: 1.04–7.24). Yet, the spatial risk patterns across the country remained largely unchanged, though with a slight increase in the posterior EPs (>95%), suggesting the presence of high‐risk clusters (RR > 1) in the Karamoja, Bunyoro, Buganda, and Ankole subregions (Supporting Information: File 9). The location of low‐risk clusters (posterior probability < 20%) and districts with the greatest uncertainty (posterior probability ~50%) was similar to the model without vaccination (BHM spatial Poisson—district).

### 3.6. Model Checks and Sensitivity Analysis

Model diagnostics suggested good overall fit and predictive performance (Supporting Information: File 10), with the data being reasonably well captured by the model results. The scatterplot of the posterior means of the predictive distributions against the observed outbreak counts shows that the data are reasonably well‐explained by the final model (*r* = 0.99). Spatial variation in local DIC values indicated heterogeneity in district‐level model fit and uncertainty, suggesting that the model performed better in some areas than others. Districts with higher local DIC values, indicating comparatively poorer model fit, were predominantly located in peripheral areas within the Buganda, West Nile, Bukedi, and Busoga subregions. The local DIC mapping broadly corresponds with the findings from the EPs (posterior probability < 50%) and higher spatial excess risk. Overall, these patterns may reflect variation in the spatial distribution of covariates, missing or unmeasured epidemiological covariables, differences in reporting, or genuinely atypical districts relative to what was captured by the best model.

Estimated covariate effects and spatial risk patterns were robust to alternative prior assumptions on the magnitude of spatial variation for the best model (BHM spatial Poisson—district). Lastly, as part of the sensitivity analysis, an ecological regression model using the ZIP distribution, identified as the second‐best spatial structure for the data during earlier stages of the analysis (Supporting Information: File 5), was built. This model (BHM spatial ZIP—district) suggested a geographical pattern of FMD risk similar to that observed with the selected BHM spatial Poisson—district model, with only a small proportion of observations (mean = 0.019) likely attributable to zero inflation, thereby supporting the final model choice (BHM spatial Poisson—district). The ZIP model also provided similar evidence of reduced risk with higher human density and in more deprived areas, whereas higher risk was associated with increased cattle density and decreased EVI in the dry period. The 95% CrIs remained almost unchanged relative to those reported in the Poisson model. Results for the ZIP ecological regression and spatial mapping are provided in Supporting Information: File 11.

## 4. Discussion

By explicitly accounting for spatial dependence, contextual drivers, and uncertainty within a Bayesian framework, the results of this analysis raised four key points: (i) the cattle corridor represents a high‐risk zone for FMD, (ii) viral circulation may occur undetected across large areas of the country, (iii) several epidemiological factors can meaningfully influence spatial risk patterns for FMD, and (iv) vaccination coverage largely reflects the distribution of outbreaks for the studied period. Together, these findings highlight the importance of a robust surveillance system and the need of high‐quality epidemiological data to support risk‐based prioritization of disease control efforts and guide resource allocation, including targeted mass vaccination, as an evidence‐based pathway for FMD control.

### 4.1. Risk Mapping and Disease Surveillance

Districts located along the cattle corridor were the main hotspots for FMD outbreaks, suggesting either higher transmission efficiency, recurrent introductions, or better disease detection [71]. Apart from hosting the largest cattle population in the country across diverse livestock systems, agroecological zones, and social contexts, the cattle corridor plays a key role in livestock trade dynamics [47, 72], highlighting its economic significance and potential role in epidemic spread. Persistent high‐risk clusters across South Buganda, Ankole, and the Karamoja subregions have been documented over the years. The Ugandan‐Tanzanian border is a well‐recognized FMD hotspot and has been heavily targeted for disease control, supported by epidemiological research aimed at understanding viral diversity, socio‐epidemiological drivers, and economic impacts of FMD [4, 21, 73–77]. In parallel, the Karamoja region, characterized by a regular occurrence of FMD [77, 78], has implemented extensive NGO‐supported vaccination programs to reduce disease transmission and mitigate its effects on the livestock‐dependent local communities.

Lower‐risk areas were also identified along the periphery of Uganda, particularly in the north, northwest, and southwest, where agropastoral smallholders and subsistence mixed farming systems are more prevalent. However, the unexpectedly low rates of disease reporting, together with elevated spatial excess risk and lower EPs (<20%) in some regions, point to potential gaps in outbreak detection [65]. This interpretation aligns with prior evidence of circulation of serotypes O, A, SAT‐1, SAT‐2, and SAT‐3 across the country over the past decade, raising the possibility of sustained viral activity and ongoing transmission, facilitated by low vaccination coverage, extensive livestock trade, and heterogeneous implementation of biosecurity and other disease‐prevention measures across farms and livestock systems [2, 11, 27, 28, 76, 79–81]. For instance, despite few outbreak reports in eastern Uganda, phylogeographic models suggest that this area may serve as a source for serotype O, with an important role in the dissemination of this serotype across the country [11]. Similarly, susceptible species in districts in western Uganda show substantial evidence of disease exposure [76, 82, 83]. These findings, together with WOAH outbreak reports from subsequent years (2020–2025), provide an indication that FMD risk extends beyond the traditional cattle corridor boundaries, with major epidemic waves recently documented in districts previously considered to be at lower risk [84].

Developing and interpreting spatial risk patterns and predictive models over longer temporal horizons remains challenging [15, 26]. True risk heterogeneity, reporting inconsistencies, and variability in the implementation of disease prevention and control measures create imbalances that have been identified as major limitations for reliable temporal risk assessment and modeling [15, 26, 85]. Although methodological advances to address shifting outbreak distributions and uncertainty are emerging, progress has so far been largely confined to binary risk classification through machine learning approaches [68]. In this context, Bayesian inference provides a valuable framework for quantitative risk estimation and for supporting evidence‐based action [29, 30, 70].

A reliable and integrated surveillance system is critical for epidemic intelligence, allowing effective planning through timely outbreak detection and response. Event‐based surveillance systems that rely on disease reporting by local farming communities, in place for FMD in most endemic countries, depend on high levels of acceptability, local ownership, collaboration, and effective communication to function effectively [86, 87]. However, lengthy livestock market closures, movement bans, and delays in the implementation of emergency vaccination following outbreak reports can massively disrupt the dynamics of livestock trade, impact farmer livelihoods, and ultimately reduce engagement with official surveillance systems [2, 4, 27]. This is particularly the case for smallholder farmers, for whom the long‐term economic effects of reporting outbreaks often outweigh the immediate health and productivity impacts of the disease [27, 85, 88]. Addressing the operational limitations of the surveillance system and building trust in the outbreak response are essential for improving data quality, which in turn supports more robust modeling outputs to inform strategic animal health decision‐making and resource allocation. Effective FMD control also requires sustained investment in technical and operational capacity, including mass vaccination campaigns with vaccines matched to circulating strains, improved biosecurity, and enhanced traceability of livestock movements [48, 89]. These measures are critical for reducing transmission and progressing towards national and regional elimination goals.

### 4.2. Eco‐Epidemiological Drivers, Vaccination Coverage, and Outbreak Risk

The association between eco‐epidemiological factors and outbreak risk points to complex system‐level interactions underlying outbreak dynamics, potential limitations on the performance of disease surveillance at a broader spatial scale, or both. Vegetation indices such as the EVI are sensitive indicators of vegetation growth and agricultural drought and have been linked to infectious disease emergence and transmission through multiple pathways. Lower EVI may indicate increased climate vulnerability among agropastoral communities during the dry season, with implications on livestock herding, animal movement, and husbandry practices [37, 90]. Reduced vegetation and grass availability may also trigger livestock sales during periods of feed and cash shortages, particularly during the dry season [47, 91, 92]. Similar dynamics have been described in the Uganda‐Tanzania border and in the Karamoja region [74, 75].

More broadly, beyond environmental influences on animal distribution and adaptive human responses to outbreaks, several epidemiological factors have been described as “conductors” or “resistors” of viral diffusion through their role in facilitating or impeding FMDV transmission. Cattle‐dense areas may act as sources or reservoirs of infection, while locations near livestock markets can facilitate and potentially amplify viral transmission [11, 12]. In contrast, areas characterized by lower rainfall and lower cattle and crop densities often constitute at‐risk sinks [11, 16]. Consistent with this pattern, extensive serosurveys conducted in Uganda have linked anti‐NSP FMDV seropositivity to cattle‐dense areas with lower rainfall [77]. Alternatively, ecological‐level factors may influence outbreak patterns through their impacts on the environmental persistence of FMDV [93] or indirectly by hampering the provision of veterinary services, including disease surveillance and response capacity.

Since a disadvantaged socioeconomic position is a well‐established driver of infectious disease incidence [94], the negative association between poorer living standards and outbreak risk is somewhat unexpected. Deprivation, as defined here, is a multidimensional measure encompassing education, health, living standards, employment, and financial situation and therefore captures broader livelihood hardship rather than monetary poverty alone. While the two often overlap in Uganda, deprivation is more sensitive to underlying inequalities and structural vulnerabilities that can influence the exposure to and spread of infectious diseases [95]. The lower observed risk in highly deprived areas may also reflect limitations on the performance of community‐based surveillance systems [86], consistent with the inverse association observed between human population density and outbreak risk. Although the mechanisms underlying this relationship cannot be fully disentangled through area‐level spatial risk modeling, geographical accessibility likely plays a role, given its influence on disease burden and reporting [96]. Access to animal health services and engagement with surveillance may be lower in rural areas compared to urban and peri‐urban settings, potentially contributing to differences in disease reporting.

Vaccination against FMD can be used to mitigate the clinical and economic impacts of the outbreaks or to progressively reduce viral circulation [48]. In Uganda, the observed association between higher mean vaccination coverage and increased outbreak risk is consistent with the predominantly reactive deployment of vaccines [39], implemented as part of emergency response packages alongside movement restrictions and market closures to curb the outbreaks. This approach aligns with activities under the Progressive Control Pathway–PCP‐FMD Stage 2 [6, 48]. Factors such as geographical scale, timeliness, and immunogenicity are critical to the effectiveness of emergency vaccination [39, 48]. High‐potency and adequately matched vaccines can provide the rapid onset of protection required during outbreaks; however, gaps in the immunogenicity of local FMD vaccines can compromise their effectiveness [39]. Together with logistical constraints, variable levels of vaccine acceptability, and delays in emergency vaccination responses [27], all these factors further undermine the perceived value of vaccination programs in the context of recurrent FMD epidemics. Therefore, proactive mass vaccination strategies combined with risk‐based prioritization may provide a pathway towards more sustainable, long‐term control feasible under resource constraints [30].

### 4.3. Caveats of the Modeling Approach, Model Interpretation, and Future Perspectives

BHMs allow uncertainty and population connectivity to be explicitly incorporated into outbreak risk estimations. Although space‐adjacency structures are widely used in small‐area disease mapping to represent interactions between neighboring populations, limitations arise from heterogeneity in area sizes and extensions of shared boundaries [35]. Assigning weights based on geographic proximity can substantially influence risk estimates, particularly where limited disease detection occurs across extensive areas. Finer spatial resolution in Bayesian risk mapping can be achieved by incorporating point‐geolocated data, including livestock movement data or outputs from phylogeographic models to infer population connectivity [29, 70] or through artificial intelligence methods for risk estimation [68, 97, 98]. Regardless of the methodological approach, acquiring data at the level of spatial granularity required for such modeling remains challenging and is currently not feasible in many FMD‐endemic settings [15, 89]. In Uganda, new tools are being developed to improve the accuracy and timeliness of relevant data for disease modeling, including enhanced livestock movement information [47, 99]. Advances in outbreak serotyping and sequencing [100], which at present largely occur outside official surveillance systems through academic research [11, 12, 21], will also unlock the possibility of serotype‐specific models [65]. The latter are particularly relevant considering that FMD endemicity may be driven by successive epidemic waves of different serotypes rather than permanent disease circulation [8].

Spatiotemporal models allow a more comprehensive understanding of localized disease dynamics by accounting for temporal autocorrelation and exploring if outbreak risk is associated with temporal shifts in epidemiological drivers [101]. Although previous studies suggest seasonal patterns of the outbreaks with peaks during the dry season in Uganda and elsewhere [15, 25, 27, 38], the unbalanced nature of outbreak data and delays in disease reporting motivated the use of a spatial rather than a spatiotemporal modeling framework. Nonetheless, robust spatial or spatiotemporal disease risk modeling and mapping remain essential for assessing shifts in disease distribution, investigating reporting patterns, generating hypotheses regarding the role of structural factors on disease risk, and supporting decision‐making by informing the conceptualization and geographical prioritization of disease prevention and control strategies in complex epidemiological systems [35, 36].

Built within a Bayesian framework and using areal data for probabilistic risk estimation [35, 36], the model presented here is not intended to provide direct evidence of transmission mechanisms or of the specific processes driving epidemic waves at the level of individual hosts, between farms, or across the wider livestock market system. Rather, it highlights large‐scale ecological associations that may underlie the observed spatial patterns for the outbreaks. These epidemiological factors warrant further investigation when more granular spatiotemporal data become available for epidemiological modeling [102] and through field‐based studies conducted across livestock systems. Such work forms part of multiscale research aimed at understanding the pathogen and disease dynamics at molecular, host, and population levels [9, 13, 103–105]. Future work should build upon high‐level epidemiological findings to inform the design of empirical studies and meta‐population models aimed at unraveling the mechanisms underlying FMDV transmission dynamics across Uganda. In parallel, further research should evaluate the performance and challenges of existing animal health surveillance systems through mixed‐method approaches for a better contextualization of modeling outputs [106, 107].

Despite its limitations, this model suggests that outbreak risk patterns were driven predominantly by spatial variation, a common finding in small‐area disease mapping [70]. Although cattle and human population density, deprivation, vegetation index, and mean vaccination coverage were associated with spatial heterogeneity in risk, additional epidemiological factors outside the scope of this study or not captured by readily available data may also influence FMD outbreak risk. These may include the role of wildlife, which was not examined in this model. At present, the involvement of African buffaloes on large‐scale FMD epidemics in Uganda remains unclear, with prior studies reporting mixed evidence for this association [26, 28, 77, 108]. This highlights the need for integrated epidemiological studies capable of capturing the broader system within which FMDV circulates in endemic settings.

## 5. Conclusions

This study highlights that the cattle corridor remains a critical zone for FMD in Uganda, where drought‐prone and cattle‐dense landscapes may favor the introduction and transmission of FMDV, triggering outbreaks of variable geographic extent. In the light of previous scientific evidence, our findings also raise the possibility of undetected viral circulation across extensive areas of the country, likely linked to limitations in the performance of community‐based disease surveillance and low vaccination coverage in those areas. A robust, sensitive, and cost‐effective surveillance system would enable animal health authorities to better guide the strategic allocation of financial, technical, and logistical resources available for disease prevention and response. Considering eco‐epidemiological aspects and spatial variability in disease risk is essential to design evidence‐informed strategies that tackle the underlying epidemiological drivers of the outbreaks. Through an iterative process involving better data, continuous risk modeling, and informed allocation of animal health resources, Uganda can support steadier progress along the PCP‐FMD pathway towards disease elimination.

## Author Contributions

Lina González Gordon was responsible for conceptualization, data curation, methodology, formal analysis, visualization, interpretation of results, and drafting the original manuscript. Dennis Muhanguzi handled aspects of data acquisition, provision of local resources, interpretation of results, and manuscript review and editing. Adrian Muwonge contributed to the data acquisition and interpreting results, supervision, and reviewing and editing the manuscript. Sylvester Ochwo, Noelina Nantima, Rose Ademun, and Norbert Frank Mwiine collected and curated the outbreak data. Lisa Boden reviewed and edited the final manuscript. Barend Mark de C. Bronsvoort supervised the work, overseeing its conceptualization and data acquisition, formal analysis, and interpretation, and contributed to manuscript revision and editing. Thibaud Porphyre provided supervision; guided the conceptualization, data acquisition, and methodology; provided interpretative insights; and contributed to reviewing and editing the manuscript.

## Acknowledgments

The authors used Grammarly (accessed December/January 2022) and GPT‐5 (accessed January/February 2026) to improve the clarity and flow of the text, all of which were written by the authors.

## Funding

Lina González Gordon was supported by the University of Edinburgh through the Principal’s Career Development and Edinburgh Global Scholarships and the Royal Society of Edinburgh Saltire Early Career Fellowship for this research. Adrian Muwonge is a Chancellor’s Fellow at the Roslin Institute, and his time was paid for by core funding and as a Future Leader Fellow funded by BBSRC (Grant BB/P007767/1) and Welcome Trust ISSF3 (Welcome Trust ISSF3). Thibaud Porphyre would like to thank the French National Research Agency and Boehringer Ingelheim Animal Health France for support through the IDEXLYON project (Grant ANR‐16‐IDEX‐0005) and the Industrial Chair in Veterinary Public Health, as part of the VPH Hub in Lyon. Barend Mark de C. Bronsvoort is supported by the BBSRC Institute Strategic Program Grant (Grant BBS/E/RL/230002D).

## Disclosure

All AI‐assisted suggestions for improved text were carefully reviewed to ensure that the meaning and accuracy were kept as originally intended. All authors read and approved the final manuscript.

## Ethics Statement

This study used outbreak data routinely collected for disease surveillance and analyzed with permission from the Department of Animal Health, Ministry of Agriculture, Animal Industry and Fisheries (MAAIF), Uganda.

## Conflicts of Interest

Noelina Nantima, Rose Ademun, and Sylvester Ochwo were government veterinary officials at the time of data collection and analysis. The other authors declare no conflicts of interest.

## Supporting Information

Additional supporting information can be found online in the Supporting Information section.

## Supporting information


**Supporting Information** Additional tables, figures, and supplementary material relevant to the analyses presented in this manuscript.

## Data Availability

The data that support the findings of this study are available from the Department of Animal Health, Ministry of Agriculture, Animal Industry and Fisheries (Uganda). Restrictions apply to its availability as it was used under license for this analysis.

## References

[1] Paton D. J. , Gubbins S. , and King D. P. , Understanding the Transmission of Foot-and-Mouth Disease Virus at Different Scales, Current Opinion in Virology. (2018) 28, 85–91, 10.1016/j.coviro.2017.11.013. 29245054

[2] Ademun A. R. , Effect of Foot and Mouth Disease in Cattle on Household Income in Selected Agro-Pastoral and Pastoral Areas of Uganda, 2012, Makerere University.

[3] Baluka S. A. , Economic Effects of Foot and Mouth Disease Outbreaks Along the Cattle Marketing Chain in Uganda, Veterinary World. (2016) 9, no. 6, 544–553, 10.14202/vetworld.2016.544-553. 27397974 PMC4937042

[4] Kerfua S. D. , Railey A. F. , and Marsh T. L. , Household Production and Consumption Impacts of Foot and Mouth Disease at the Uganda-Tanzania Border, Frontiers in Veterinary Science. (2023) 10, 10.3389/fvets.2023.1156458, 1156458. 37342624 PMC10277485

[5] Knight-Jones T. J. D. , McLaws M. , and Rushton J. , Foot-and-Mouth Disease Impact on Smallholders—What Do We Know, What Don’t We Know and How Can We Find Out More?, Transboundary and Emerging Diseases. (2017) 64, no. 4, 1079–1094, 10.1111/tbed.12507. 27167976 PMC5516236

[6] FAO, OIE, GF-TADs, and EU-FMD , The Progressive Control Pathway for Foot and Mouth Disease Control (PCP-FMD), 2018, http://www.fao.org/3/CA1331EN/ca1331en.pdf.

[7] FAO and WOAH , The Global Foot-and-Mouth-Disease Control Strategy: Strenthening Animal Health Systems Through Improved Control of Major Diseases, 2012.

[8] Casey-Bryars M. , Reeve R. , and Bastola U. , et al.Waves of Endemic Foot-and-Mouth Disease in Eastern Africa Suggest Feasibility of Proactive Vaccination Approaches, Nature Ecology & Evolution. (2018) 2, no. 9, 1449–1457, 10.1038/s41559-018-0636-x. 30082738

[9] Woldemariyam F. T. , Kariuki C. K. , and Kamau J. , et al.Epidemiological Dynamics of Foot-and-Mouth Disease in the Horn of Africa: The Role of Virus Diversity and Animal Movement, Viruses. (2023) 15, no. 4, 10.3390/v15040969. PMC10143177 37112947

[10] Makau D. N. , Arzt J. , and VanderWaal K. , Tracing the Spread and Phylogeography of Foot-and-Mouth Disease Virus Across East and the Horn of Africa, Virus Evolution. (2025) 11, no. 1, 10.1093/ve/veaf073, veaf073. 41069416 PMC12507016

[11] Munsey A. , Mwiine F. N. , and Ochwo S. , et al.Ecological and Anthropogenic Spatial Gradients Shape Patterns of Dispersal of Foot-and-Mouth Disease Virus in Uganda, Pathogens. (2022) 11, no. 5, 10.3390/pathogens11050524. PMC9143568 35631045

[12] Munsey A. , Mwiine F. N. , and Ochwo S. , et al.Phylogeographic Analysis of Foot-and-Mouth Disease Virus Serotype O Dispersal and Associated Drivers in East Africa, Molecular Ecology. (2021) 30, no. 15, 3815–3825, 10.1111/mec.15991. 34008868

[13] Di Nardo A. , Ferretti L. , and Wadsworth J. , et al.Evolutionary and Ecological Drivers Shape the Emergence and Extinction of Foot-and-Mouth Disease Virus Lineages, Molecular Biology and Evolution. (2021) 38, no. 10, 4346–4361, 10.1093/molbev/msab172. 34115138 PMC8476141

[14] Compston P. , Limon G. , Sangula A. , Onono J. , King D. P. , and Häsler B. , Understanding What Shapes Disease Control: An Historical Analysis of Foot-and-Mouth Disease in Kenya, Preventive Veterinary Medicine. (2021) 190, 10.1016/j.prevetmed.2021.105315, 105315. 33735817

[15] González Gordon L. , Porphyre T. , Muhanguzi D. , Muwonge A. , Boden L. , and Bronsvoort B. M. D. C. , A Scoping Review of Foot-and-Mouth Disease Risk, Based on Spatial and Spatio-Temporal Analysis of Outbreaks in Endemic Settings, Transboundary and Emerging Diseases. (2022) 69, no. 6, 3198–3215, 10.1111/tbed.14769. 36383164 PMC10107783

[16] Duchatel F. , Bronsvoort B. M. D. C. , and Lycett S. , Phylogeographic Analysis and Identification of Factors Impacting the Diffusion of Foot-and-Mouth Disease Virus in Africa, Frontiers in Ecology and Evolution. (2019) 7, 10.3389/fevo.2019.00371.

[17] Souley Kouato B. , De Clercq K. , and Abatih E. , et al.Review of Epidemiological Risk Models for Foot-and-Mouth Disease: Implications for Prevention Strategies With a Focus on Africa, PLoS One. (2018) 13, no. 12, 10.1371/journal.pone.0208296, e0208296. 30543641 PMC6292601

[18] Card C. , Epp T. , and Lem M. , Exploring the Social Determinants of Animal Health, Journal of Veterinary Medical Education. (2018) 45, no. 4, 437–447, 10.3138/jvme.0317-047r. 30285599

[19] Xia S. , Zhou X.-N. , and Liu J. , Systems Thinking in Combating Infectious Diseases, Infectious Diseases of Poverty. (2017) 6, no. 1, 10.1186/s40249-017-0339-6. PMC5594605 28893320

[20] Kasambula L. , Belsham G. J. , Siegismund H. R. , Muwanika V. B. , Ademun-Okurut A. R. , and Masembe C. , Serotype Identification and VP1 Coding Sequence Analysis of Foot-and-Mouth Disease Viruses From Outbreaks in Eastern and Northern Uganda in 2008/9, Transboundary and Emerging Diseases. (2012) 59, no. 4, 323–330, 10.1111/j.1865-1682.2011.01276.x. 22117844

[21] Kerfua S. D. , Shirima G. , and Kusiluka L. , et al.Low Topotype Diversity of Recent Foot-and-Mouth Disease Virus Serotypes O and A From Districts Located Along the Uganda and Tanzania Border, Journal of Veterinary Science. (2019) 20, no. 2, 10.4142/jvs.2019.20.e4, 1119451. PMC6441803 30944527

[22] Mwiine F. N. , Velazquez-Salinas L. , and Ahmed Z. , et al.Serological and Phylogenetic Characterization of Foot and Mouth Disease Viruses From Uganda during Cross-Sectional Surveillance Study in Cattle Between 2014 and 2017, Transboundary and Emerging Diseases. (2019) 66, no. 5, 2011–2024, 10.1111/tbed.13249. 31127983

[23] Ayebazibwe C. , Mwiine F. N. , and Balinda S. N. , et al.Antibodies against Foot-and-Mouth Disease (FMD) Virus in African Buffalos (*Syncerus caffer*) in Selected National Parks in Uganda (2001-2003, Transboundary and Emerging Diseases. (2010) 57, 286–292, 10.1111/j.1865-1682.2010.01147.x. 20561289

[24] Balinda S. N. , Belsham G. J. , Masembe C. , Sangula A. K. , Siegismund H. R. , and Muwanika V. B. , Molecular Characterization of SAT 2 Foot-and-Mouth Disease Virus From Post-Outbreak Slaughtered Animals: Implications for Disease Control in Uganda, Epidemiology and Infection. (2010) 138, no. 8, 1204–1210, 10.1017/S0950268809991427. 20003615

[25] Okello J. , Okello W. , and Muhanguzi S. , et al.Spatial and Temporal Distribution of Foot and Mouth Disease in Cattle in Uganda From 2010–2021 (A Retrospective Study), 2022, https://www.researchsquare.com/article/rs-2013492/v1.

[26] Kapalaga G. , Kivunike F. N. , and Kerfua S. , et al.A Unified Foot and Mouth Disease Dataset for Uganda: Evaluating Machine Learning Predictive Performance Degradation Under Varying Distributions, Frontiers in Artificial Intelligence. (2024) 7, 10.3389/frai.2024.1446368, 1446368. 39144542 PMC11322090

[27] Muleme M. , Barigye R. , Khaitsa M. L. , Berry E. , Wamono A. W. , and Ayebazibwe C. , Effectiveness of Vaccines and Vaccination Programs for the Control of Foot-and-Mouth Disease in Uganda, 2001-2010, Tropical Animal Health and Production. (2013) 45, no. 1, 35–43, 10.1007/s11250-012-0254-6. 22956440

[28] Ayebazibwe C. , Tjørnehøj K. , and Mwiine F. N. , et al.Patterns, Risk Factors and Characteristics of Reported and Perceived Foot-and-Mouth Disease (FMD) in Uganda, Tropical Animal Health and Production. (2010) 42, no. 7, 1547–1559, 10.1007/s11250-010-9605-3. 20526861

[29] Gunasekara U. , Bertram M. R. , and Van Long N. , et al.Phylogeography as a Proxy for Population Connectivity for Spatial Modeling of Foot-and-Mouth Disease Outbreaks in Vietnam, Viruses. (2023) 15, no. 2, 10.3390/v15020388. PMC9958845 36851602

[30] Gunasekera U. , Biswal J. K. , and Machado G. , et al.Impact of Mass Vaccination on the Spatiotemporal Dynamics of FMD Outbreaks in India, 2008-2016, Transboundary and Emerging Diseases. (2022) 69, no. 5, e1936–e1950, 10.1111/tbed.14528. 35306749 PMC9790522

[31] Hossain M. M. and Lawson A. B. , Cluster Detection Diagnostics for Small Area Health Data: With Reference to Evaluation of Local Likelihood Models, Statistics in Medicine. (2006) 25, no. 5, 771–786, 10.1002/sim.2401. 16453370

[32] Lawson A. B. and Zhou H. , Spatial Statistical Modeling of Disease Outbreaks With Particular Reference to the UK Foot and Mouth Disease (FMD) Epidemic of 2001, Preventive Veterinary Medicine. (2005) 71, no. 3-4, 141–156, 10.1016/j.prevetmed.2005.07.002. 16188334

[33] Singer B. J. , Thompson R. N. , and Bonsall M. B. , Evaluating Strategies for Spatial Allocation of Vaccines Based on Risk and Centrality, Journal of the Royal Society Interface. (2022) 19, no. 187, 10.1098/rsif.2021.0709, 20210709. 35167774 PMC8847001

[34] Dlamini W. M. , Dlamini S. N. , Mabaso S. D. , and Simelane S. P. , Spatial Risk Assessment of an Emerging Pandemic Under Data Scarcity: A Case of COVID-19 in Eswatini, Applied Geography. (2020) 125, 10.1016/j.apgeog.2020.102358, 102358. 33132463 PMC7586938

[35] Lawson A. B. , Bayesian Disease Mapping: Hierarchical Modeling in Spatial Epidemiology, 2018, Chapman & Hall, 10.1201/9781351271769.

[36] Lawson A. B. , Using R for Bayesian Spatial and Spatio-Temporal Health Modeling, 2021, Chapman and Hall/CRC, 10.1201/9781003043997.

[37] Turner M. D. and Schlecht E. , Livestock Mobility in Sub-Saharan Africa: A Critical Review, Pastoralism. (2019) 9, no. 1, 1–15, 10.1186/s13570-019-0150-z.

[38] Byamukama B. , Amin A. , Mwiine F. N. , and Ekiri A. B. , Epidemiology and Control Strategies for Foot-and-Mouth Disease in Livestock and Wildlife in Uganda: Systematic Review, Veterinary Research Communications. (2025) 49, no. 4, 10.1007/s11259-025-10791-z. PMC12170765 40522510

[39] Kerfua S. D. , Haydon D. T. , and Wilsden G. , et al.Evaluation of Commercial Quadrivalent Foot-and-Mouth Disease Vaccines Against East African Virus Strains Reveals Limited Immunogenicity and Duration of Protection, Vaccine. (2024) 42, no. 26, 10.1016/j.vaccine.2024.126325, 126325. 39270355

[40] Nimusiima A. , Basalirwa , and Majaliwa J. G. M. , et al.Nature and Dynamics of Climate Variability in the Uganda Cattle Corridor, African Journal of Environmental Science and Technology. (2013) 7, no. 8, 770–782, 10.5897/AJEST2013.1435.

[41] Busetto L. and Ranghetti L. , MODIStsp: An R Package for Automatic Preprocessing of MODIS Land Products Time Series, Computers & Geosciences. (2016) 97, 40–48, 10.1016/j.cageo.2016.08.020.

[42] Didan K. , MODIS/Terra Vegetation Indices 16-Day L3 Global 250m SIN Grid V061 [Data Set], 2021, NASA EOSDIS Land Processes DAAC.

[43] Gilbert M. , Nicolas G. B. , and Cinardi G. , et al.Global Distribution Data for Cattle, Buffaloes, Horses, Sheep, Goats, Pigs, Chickens and Ducks in 2010, Scientific Data. (2018) 5, no. 1, 10.1038/sdata.2018.227, 180227. 30375994 PMC6207061

[44] UBOS , Population Projects by District, 2015–2021, 2022, https://www.ubos.org/explore-statistics/20/UBOSstandsforUgandaBureauofStatistics.

[45] Development Initiatives , Spotlight on Uganda, 2022, https://devinit.github.io/data/datasets/?nav=header.

[46] Wang X. , Rafa M. , Moyer J. D. , Li J. , Scheer J. , and Sutton P. , Estimation and Mapping of Sub-National GDP in Uganda Using NPP-VIIRS Imagery, Remote Sensing. (2019) 11, no. 2, 10.3390/rs11020163.

[47] González-Gordon L. , Porphyre T. , and Muwonge A. , et al.Identifying Target Areas for Risk-Based Surveillance and Control of Transboundary Animal Diseases: A Seasonal Analysis of Slaughter and Live-Trade Cattle Movements in Uganda, Scientific Reports. (2023) 13, no. 1, 10.1038/s41598-023-44518-4, 18619. 37903814 PMC10616094

[48] Ferrari G. , Foot and Mouth Disease Vaccination and Post-Vaccination Monitoring: Guidelines, 2016, Food and Agriculture Organization of the United Nations World Organisation for Animal Health.

[49] Lindgren F. and Rue H. , Bayesian Spatial Modelling With R-INLA, Journal of Statistical Software. (2015) 63, no. 19, 1–25, 10.18637/jss.v063.i19.

[50] Rue H. , Martino S. , and Chopin N. , Approximate Bayesian Inference for Latent Gaussian Models by Using Integrated Nested Laplace Approximations, Journal of the Royal Statistical Society Series B: Statistical Methodology. (2009) 71, no. 2, 319–392, 10.1111/j.1467-9868.2008.00700.x.

[51] Blangiardo M. and Cameletti M. , Spatial and Spatio-Temporal Bayesian Models With R-INLA/by Marta Blangiardo and Michela Cameletti, 2015, John Wiley and Sons, Inc..

[52] Coly S. , Garrido M. , Abrial D. , Yao A.-F. , and Siettos C. , Bayesian Hierarchical Models for Disease Mapping Applied to Contagious Pathologies, PLoS ONE. (2021) 16, no. 1, 10.1371/journal.pone.0222898, e0222898. 33439868 PMC7806170

[53] Wickham H. , François R. , Henry L. , Müller K. , and Vaughan D. , dplyr: A Grammar of Data Manipulation, 2026, R package version 1.2.0, https://CRAN.R-project.org/package=dplyr, 10.32614/CRAN.package.dplyr.

[54] Wickham H. , Ggplot2: Elegant Graphics for Data Analysis, 2016, Springer-Verlag.

[55] Wickham H. , Vaughan D. , and Girlich M. , tidyr: Tidy Messy Data, 2025, R package version 1.3.2, https://CRAN.R-project.org/package=tidyr, 10.32614/CRAN.package.tidyr.

[56] Besag J. , York J. , and Mollié A. , Bayesian Image Restoration, With Two Applications in Spatial Statistics, Annals of the Institute of Statistical Mathematics. (1991) 43, no. 1, 1–20, 10.1007/BF00116466.

[57] Wei T. and Simko V. , R Package ’Corrplot’: Visualization of a Correlation Matrix (Version 0.95), 2024, https://github.com/taiyun/corrplot.

[58] Zuur A. F. , Mixed Effects Models and Extensions in Ecology With R, 2009, 1 edition, Springer, New York, 10.1007/978-0-387-87458-6.

[59] O’Hara R. B. and Sillanpää M. J. , A Review of Bayesian Variable Selection Methods: What, How and which, Bayesian Analysis. (2009) 4, 85–117.

[60] Lawson A. B. , Carroll R. , Faes C. , Kirby R. S. , Aregay M. , and Watjou K. , Spatiotemporal Multivariate Mixture Models for Bayesian Model Selection in Disease Mapping, Environmetrics. (2017) 28, no. 8, 10.1002/env.2465, e2465. 29230091 PMC5722237

[61] Schrödle B. , Held L. , Riebler A. , and Danuser J. , Using Integrated Nested Laplace Approximations for the Evaluation of Veterinary Surveillance Data From Switzerland: A Case-Study, Journal of the Royal Statistical Society: Series C (Applied Statistics). (2011) 60, 261–279.

[62] Spiegelhalter D. J. , Best N. G. , Carlin B. P. , and Van Der Linde A. , Bayesian Measures of Model Complexity and Fit, Journal of the Royal Statistical Society Series B: Statistical Methodology. (2002) 64, no. 4, 583–639, 10.1111/1467-9868.00353.

[63] Carroll R. , Lawson A. B. , Faes C. , Kirby R. S. , Aregay M. , and Watjou K. , Spatially-Dependent Bayesian Model Selection for Disease Mapping, Statistical Methods in Medical Research. (2018) 27, no. 1, 250–268, 10.1177/0962280215627298. 28034176 PMC5374035

[64] Richardson S. , Thomson A. , Best N. , and Elliott P. , Interpreting Posterior Relative Risk Estimates in Disease-Mapping Studies, Environmental Health Perspectives. (2004) 112, no. 9, 1016–1025, 10.1289/ehp.6740. 15198922 PMC1247195

[65] Richards K. K. , Hazelton M. L. , Stevenson M. A. , Lockhart C. Y. , Pinto J. , and Nguyen L. , Using Exceedance Probabilities to Detect Anomalies in Routinely Recorded Animal Health Data, With Particular Reference to Foot-and-Mouth Disease in Viet Nam, Spatial and Spatio-temporal Epidemiology. (2014) 11, 125–133, 10.1016/j.sste.2014.08.002. 25457601

[66] Arab A. , Spatial and Spatio-Temporal Models for Modeling Epidemiological Data With Excess Zeros, International Journal of Environmental Research and Public Health. (2015) 12, no. 9, 10536–10548, 10.3390/ijerph120910536. 26343696 PMC4586626

[67] Blasco-Moreno A. , Pérez-Casany M. , Puig P. , Morante M. , and Castells E. , What Does a Zero Mean? Understanding False, Random and Structural Zeros in Ecology, Methods in Ecology and Evolution. (2019) 10, no. 7, 949–959, 10.1111/2041-210X.13185.

[68] Kapalaga G. , Kivunike F. N. , and Kerfua S. , et al.Enhancing Random Forest Predictive Performance for Foot and Mouth Disease Outbreaks in Uganda: A Calibrated Uncertainty Prediction Approach for Varying Distributions, Frontiers in Artificial Intelligence. (2024) 7, 10.3389/frai.2024.1455331, 1455331. 39554990 PMC11564173

[69] Bernardinelli L. , Clayton D. , and Montomoli C. , Bayesian Estimates of Disease Maps: How Important are Priors?, Statistics in Medicine. (1995) 14, no. 21-22, 2411–2431, 10.1002/sim.4780142111. 8711278

[70] Madden J. M. , McGrath G. , Sweeney J. , Murray G. , Tratalos J. A. , and More S. J. , Spatio-Temporal Models of Bovine Tuberculosis in the Irish Cattle Population, 2012-2019, Spatial and Spatio-Temporal Epidemiology. (2021) 39, 10.1016/j.sste.2021.100441, 100441. 34774256

[71] Lessler J. , Azman A. S. , McKay H. S. , and Moore S. M. , What is a Hotspot Anyway?, The American Society of Tropical Medicine and Hygiene. (2017) 96, no. 6, 1270–1273, 10.4269/ajtmh.16-0427.

[72] Hasahya E. , Thakur K. , Dione M. M. , Kerfua S. D. , Mugezi I. , and Lee H. S. , Analysis of Patterns of Livestock Movements in the Cattle Corridor of Uganda for Risk-Based Surveillance of Infectious Diseases, Frontiers in Veterinary Science. (2023) 10, 10.3389/fvets.2023.1095293, 1095293. 36756309 PMC9899994

[73] Kerfua S. D. , Isubikalu P. , Ademun-Okurut R. A. , Muwanika V. B. , and Masembe C. , Molecular Characterization of Serotype O Foot-and-Mouth Disease Virus From Pigs: Implications for Multi-Species Approach to Disease Control in Uganda, African Journal of Biotechnology. (2013) 12, 2547–2552.

[74] Kerfua S. D. , Shirima G. , and Kusiluka L. , et al.Spatial and Temporal Distribution of Foot-and-Mouth Disease in Four Districts Situated Along the Uganda-Tanzania Border: Implications for Cross-Border Efforts in Disease Control, Onderstepoort Journal of Veterinary Research. (2018) 85, no. 1, e1–e8, 10.4102/ojvr.v85i1.1528. PMC6238673 30198279

[75] Mugezi I. , Kimaanga M. , and Namwabira A. , et al.Risk of Foot and Mouth Disease Spread Through Cattle Movements in Uganda, Revue Scientifique et Technique (International Office of Epizootics). (2020) 39, no. 3, 847–861, 10.20506/rst.39.3.3182. 35275131

[76] Balinda S. N. , Tjørnehøj K. , and Muwanika V. B. , et al.Prevalence Estimates of Antibodies Towards Foot-and-Mouth Disease Virus in Small Ruminants in Uganda, Transboundary and Emerging Diseases. (2009) 56, no. 9-10, 362–371, 10.1111/j.1865-1682.2009.01094.x. 19909475

[77] Munsey A. , Mwiine F. N. , and Ochwo S. , et al.Spatial Distribution and Risk Factors for Foot and Mouth Disease Virus in Uganda: Opportunities for Strategic Surveillance, Preventive Veterinary Medicine. (2019) 171, 10.1016/j.prevetmed.2019.104766, 104766. 31541845

[78] Kansiime M. , Seroprevalence and Serotypes of Foot-and-Mouth Disease Virus in Apparently Clinically Healthy Small and Large Ruminant Populations in Selected Districts of the Karamoja Sub-Region, North-Eastern Uganda, 2024, Makerere University.

[79] Namatovu A. , Tjørnehøj K. , and Belsham G. J. , et al.Characterization of Foot-and-Mouth Disease Viruses (FMDVs) From Ugandan Cattle Outbreaks During 2012-2013: Evidence for Circulation of Multiple Serotypes, PLoS One. (2015) 10, no. 2, 10.1371/journal.pone.0114811, e0114811. 25664876 PMC4321839

[80] Isubikalu P. , Masembe C. , Muwanika V. , and Ademun Okurut A. R. , Understanding the Persistence of Foot-and-Mouth Disease in Uganda: The Case of Western Uganda, 2010, https://repository.ruforum.org/system/tdf/Isubikalu.pdf?file=1&type=node&id=34739&force=.

[81] Wolff C. , Abigaba S. , and Sternberg Lewerin S. , Ugandan Cattle Farmers’ Perceived Needs of Disease Prevention and Strategies to Improve Biosecurity, BMC Veterinary Research. (2019) 15, no. 1, 10.1186/s12917-019-1961-2. PMC6588948 31226988

[82] Dhikusooka M. T. , Ayebazibwe C. , and Namatovu A. , et al.Unrecognized Circulation of SAT 1 Foot-and-Mouth Disease Virus in Cattle Herds Around Queen Elizabeth National Park in Uganda, BMC Veterinary Research. (2016) 12, no. 1, 10.1186/s12917-015-0616-1. PMC4704403 26739166

[83] Mwiine F. N. , Ayebazibwe C. , Olaho-Mukani W. , Alexandersen S. , and Tjornehoj K. , Prevalence of Antibodies Against Foot-and-Mouth Disease Virus in Cattle in Kasese and Bushenyi Districts in Uganda, International Journal of Animal and Veterinary Advances. (2010) 2, 89–96.

[84] World Organisation for Animal Health (WOAH) , World Animal Health Information System (WAHIS), 2025, WOAH, Paris, https://wahis.woah.org/.

[85] Motta P. , Garner G. , and Hòvari M. , et al.A Framework for Reviewing Livestock Disease Reporting Systems in High-Risk Areas: Assessing Performance and Perceptions Towards Foot and Mouth Disease Reporting in the Thrace Region of Greece, Bulgaria and Turkey, Transboundary and Emerging Diseases. (2019) 66, no. 3, 1268–1279, 10.1111/tbed.13143. 30734513

[86] McGowan C. R. , Takahashi E. , and Romig L. , et al.Community-Based Surveillance of Infectious Diseases: A Systematic Review of Drivers of Success, BMJ Global Health. (2022) 7, no. 8, 10.1136/bmjgh-2022-009934, e009934. PMC9396156 35985697

[87] Nansikombi H. T. , Kwesiga B. , Aceng F. L. , Ario A. R. , Bulage L. , and Arinaitwe E. S. , Timeliness and Completeness of Weekly Surveillance Data Reporting on Epidemic Prone Diseases in Uganda, 2020-2021, BMC Public Health. (2023) 23, no. 1, 10.1186/s12889-023-15534-w. PMC10072024 37016380

[88] MacPhillamy I. , Young J. , and Earp F. , et al.Foot-and-Mouth Disease Seroprevalence and Reporting Behaviours in Nine Northern Provinces in Lao PDR: The Current Situation and Challenges for Control, Transboundary and Emerging Diseases. (2021) 69, no. 2, 645–659, 10.1111/tbed.14031. 33559340

[89] GFRA , Global Foot-and-Mouth Disease Research Alliance (GFRA) Gap Analysis Report, 2022, https://go.usa.gov/xdrKh.

[90] Baluka S. A. , Hisali E. , Wasswa F. , Ocaido M. , and Mugisha A. , Socioeconomic Risk Factors Associated With Foot and Mouth Disease, and Contagious Bovine Pleuropneumonia Outbreaks in Uganda, Livestock Research for Rural Development. (2013) 25.

[91] Nanfuka S. , Mfitumukiza D. , and Egeru A. , Characterisation of Ecosystem-Based Adaptations to Drought in the Central Cattle Corridor of Uganda, African Journal of Range & Forage Science. (2020) 37, no. 4, 257–267, 10.2989/10220119.2020.1748713.

[92] Ruhangawebare G. K. , Factors Affecting the Level of Commercialization Among Cattle Keepers in the Pastoral Areas of Uganda, 2010.

[93] Mielke S. R. and Garabed R. , Environmental Persistence of Foot-and-Mouth Disease Virus Applied to Endemic Regions, Transboundary and Emerging Diseases. (2020) 67, no. 2, 543–554, 10.1111/tbed.13383. 31595659

[94] Ayorinde A. , Ghosh I. , and Ali I. , et al.Health Inequalities in Infectious Diseases: A Systematic Overview of Reviews, BMJ Open. (2023) 13, no. 4, 10.1136/bmjopen-2022-067429, e067429. PMC10083762 37015800

[95] Guerrini L. , Pfukenyi D. M. , and Etter E. , et al.Spatial and Seasonal Patterns of FMD Primary Outbreaks in Cattle in Zimbabwe Between 1931 and 2016, Veterinary Research. (2019) 50, no. 1, 10.1186/s13567-019-0690-7. PMC6760110 31551078

[96] Hierink F. , Okiro E. A. , Flahault A. , and Ray N. , The Winding Road to Health: A Systematic Scoping Review on the Effect of Geographical Accessibility to Health Care on Infectious Diseases in Low- and Middle-Income Countries, PLOS ONE. (2021) 16, no. 1, 10.1371/journal.pone.0244921, e0244921. 33395431 PMC7781385

[97] Alkhamis M. A. , Abouelhassan H. , and Alateeqi A. , et al.Predicting the Landscape Epidemiology of Foot-and-Mouth Disease in Endemic Regions: An Interpretable Machine Learning Approach, Viruses. (2025) 17, no. 10, 10.3390/v17101383, 1383. 41157653 PMC12567987

[98] Punyapornwithaya V. , Klaharn K. , Arjkumpa O. , and Sansamur C. , Exploring the Predictive Capability of Machine Learning Models in Identifying Foot and Mouth Disease Outbreak Occurrences in Cattle Farms in an Endemic Setting of Thailand, Preventive Veterinary Medicine. (2022) 207, 10.1016/j.prevetmed.2022.105706, 105706. 35863259

[99] Muwonge A. , Wee B. A. , and Mugerwa I. , et al.An Open-Source Digital Contact Tracing System Tailored to Haulage, Frontiers in Digital Health. (2023) 5, 10.3389/fdgth.2023.1199635, 1199635. 37538199 PMC10394895

[100] Shaw A. E. , Lebani K. , and González Gordon L. , et al.Universal Amplification and Sequencing of Foot-and-Mouth Disease Virus Complete Genomes Using Nanopore Technology, BMC Genomics. (2025) 26, no. 1, 10.1186/s12864-025-11938-7. PMC12372193 40846908

[101] Abellan J. J. , Richardson S. , and Best N. , Use of Space-Time Models to Investigate the Stability of Patterns of Disease, Environmental Health Perspectives. (2008) 116, no. 8, 1111–1119, 10.1289/ehp.10814. 18709143 PMC2516563

[102] Pomeroy L. W. , Bansal S. , and Tildesley M. , et al.Data-Driven Models of Foot-and-Mouth Disease Dynamics: A Review, Transboundary and Emerging Diseases. (2017) 64, no. 3, 716–728, 10.1111/tbed.12437. 26576514 PMC5205574

[103] Antonovics J. , Transmission Dynamics: Critical Questions and Challenges, Philosophical Transactions of the Royal Society B: Biological Sciences. (2017) 372, no. 1719, 10.1098/rstb.2016.0087, 20160087. PMC5352814 28289255

[104] Garabed A. R. B. , Johnson W. O. , Gill J. , Perez A. M. , and Thurmond M. C. , Exploration of Associations Between Governance and Economics and Country Level Foot- and-Mouth Disease Status by Using Bayesian Model Averaging, Journal of the Royal Statistical Society Series A: Statistics in Society. (2021) 171, 699–722.

[105] Garabed R. B. , Jolles A. , Garira W. , Lanzas C. , Gutierrez J. , and Rempala G. , Multi-Scale Dynamics of Infectious Diseases, Interface Focus. (2019) 10, no. 1, 10.1098/rsfs.2019.0118, 20190118.

[106] Martella L. , Scolamacchia F. , Mulatti P. , Cattoli G. , Lupini C. , and De Nardi M. , The Evaluation of Surveillance Systems in Veterinary Public Health: A Scoping Review on the Attributes and Assessment Frameworks Most Commonly Used, Preventive Veterinary Medicine. (2026) 253, 10.1016/j.prevetmed.2026.106879, 106879. 41962378

[107] Chenais E. , Sternberg-Lewerin S. , and Boqvist S. , et al.African Swine Fever in Uganda: Qualitative Evaluation of Three Surveillance Methods With Implications for Other Resource-Poor Settings, Frontiers in Veterinary Science. (2015) 2, 10.3389/fvets.2015.00051. PMC4673915 26664978

[108] Mwiine F. N. , Ayebazibwe C. , and Olaho-Mukani W. , et al.Serotype Specificity of Antibodies Against Foot-and-Mouth Disease Virus in Cattle in Selected Districts in Uganda, Transboundary and Emerging Diseases. (2010) 57, no. 5, 365–374, 10.1111/j.1865-1682.2010.01157.x. 20696028

