# Supplementary material for: Spatial Patterns and Epidemiological Drivers of Foot‐and‐Mouth Disease Outbreaks in Uganda
Source: Transbound Emerg Dis. 2026 Jul 21;2026:4994209. doi: 10.1155/tbed/4994209 (PMC13386121; doi:10.1155/tbed/4994209)

Additional file 1. Distribution of FMD outbreak data in Ugandan subregions. Map of Ugandan subregions; boxplots and bar charts showing the percentage of FMD outbreaks reported per subregion during the study period.

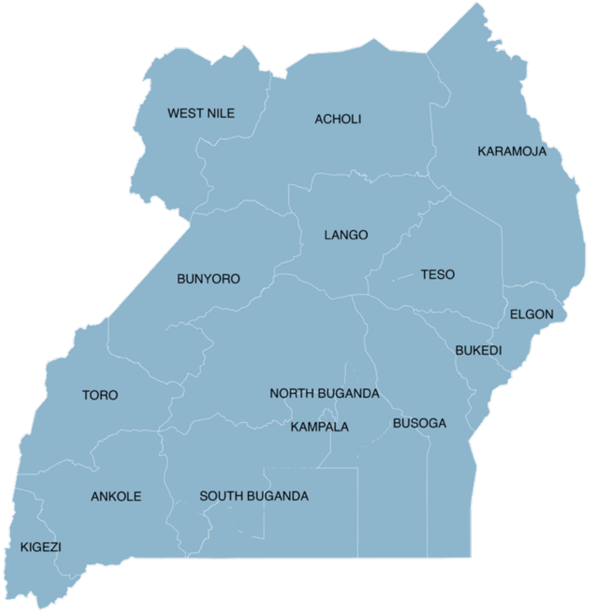

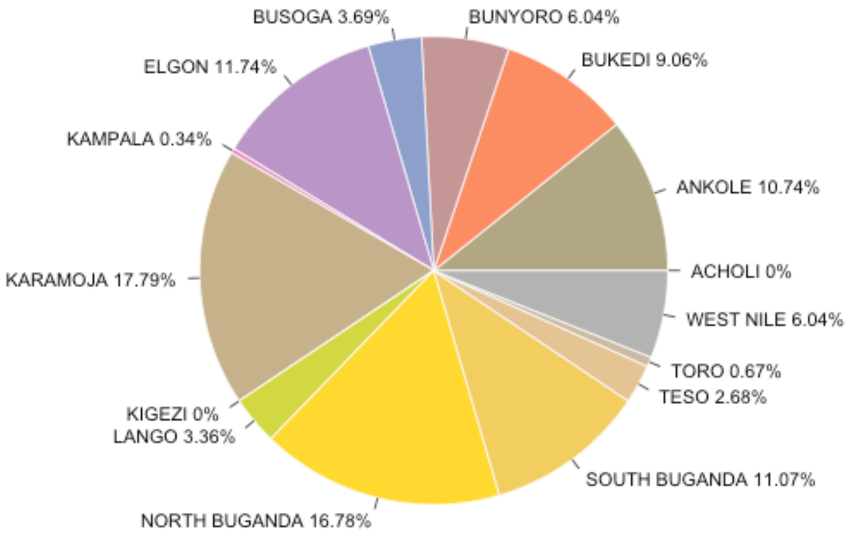

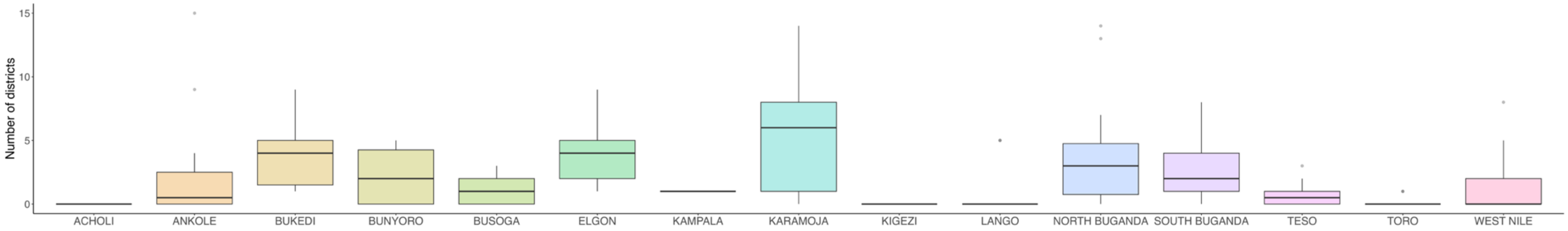

Additional file 2. Summary of variables used in FMD risk modelling. All epidemiological covariables were classified according to the framework described in González Gordon *et al.* (2022).

Risk class	Variable	Definition	Unit	Year	Source
Animal demographics	Cattle density	Number of cattle per unit area	animals/km²	2015	FAO Gridded Livestock of the World database (GLW v3.1, 2015) (Gilbert et al., 2018)
Animal demographics	Sheep density	Number of sheep per unit area	animals/km²	2015	FAO GLW v3.1, 2015
Animal demographics	Goat density	Number of goats per unit area	animals/km²	2015	FAO GLW v3.1, 2015
Animal demographics	Pig density	Number of pigs per unit area	animals/km²	2015	FAO GLW v3.1, 2015
Trade and commerce	District centrality strength	Sum of weights of all edges connected to each district, indicating cattle trade volume (in-strength = incoming animals; out-strength = outgoing animals)	number of animals	2019	MAAIF – Animal Movement Permits (González-Gordon et al., 2023)
Trade and commerce	District centrality degree	Number of trade connections per district (in-degree = incoming; out-degree = outgoing)	count	2019	MAAIF – Animal Movement Permits (González-Gordon et al., 2023)
Trade and commerce	Urban population	Population living in urban areas	%	2014	Development Initiatives, 2020
Trade and commerce	Human population density	Number of people per unit area	people/km²	2019	Uganda Bureau of Statistics (UBOS, 2023)
Trade and commerce	International border	District classification as inland or border	binary (inland/border)	2019	MAAIF – country shapefiles
Socio-economic	Deprivation score	Indicator of basic needs met for a decent standard of living (higher = better)	index score	2014	Development Initiatives, 2020
Socio-economic	Poverty estimates	Percentage of the population living below the	%	2020	Uganda Bureau of Statistics

		national poverty line			(UBOS)
Socio-economic	GDP per cap	Total economic output per person, adjusted to USD	USD	2015	Wang et al., 2019
Environmental	Enhanced Vegetation Index (EVI)	A satellite-derived index of vegetation greenness and canopy structure (range –1 to 1), calculated separately for dry and wet seasons	index value	2014–2019	MODIS product MOD13A3 (Busetto & Ranghetti, 2016; Didan, 2021)
Spatial accessibility	Road density	Density of roads per unit area (includes all types: bituminous and unsealed)	km/km²	2019	MAAIF – country shapefiles
Spatial accessibility	Proportion of water	District area containing any type of water body	%	2019	MAAIF – country shapefiles

Additional file 3. Choropleth map showing the spatial distribution of the covariates explored for ecological regression. Each covariate was mapped to a class according to the framework proposed by González-Gordon *et al.* (2022). For the Enhanced Vegetation Index (EVI), "dry season 1" corresponds to the first dry season (December–February), "dry season 2" to the second dry season (June–August), "wet season 1" to the short rains (March–May), and "wet season 2" to the long rains (September–November).

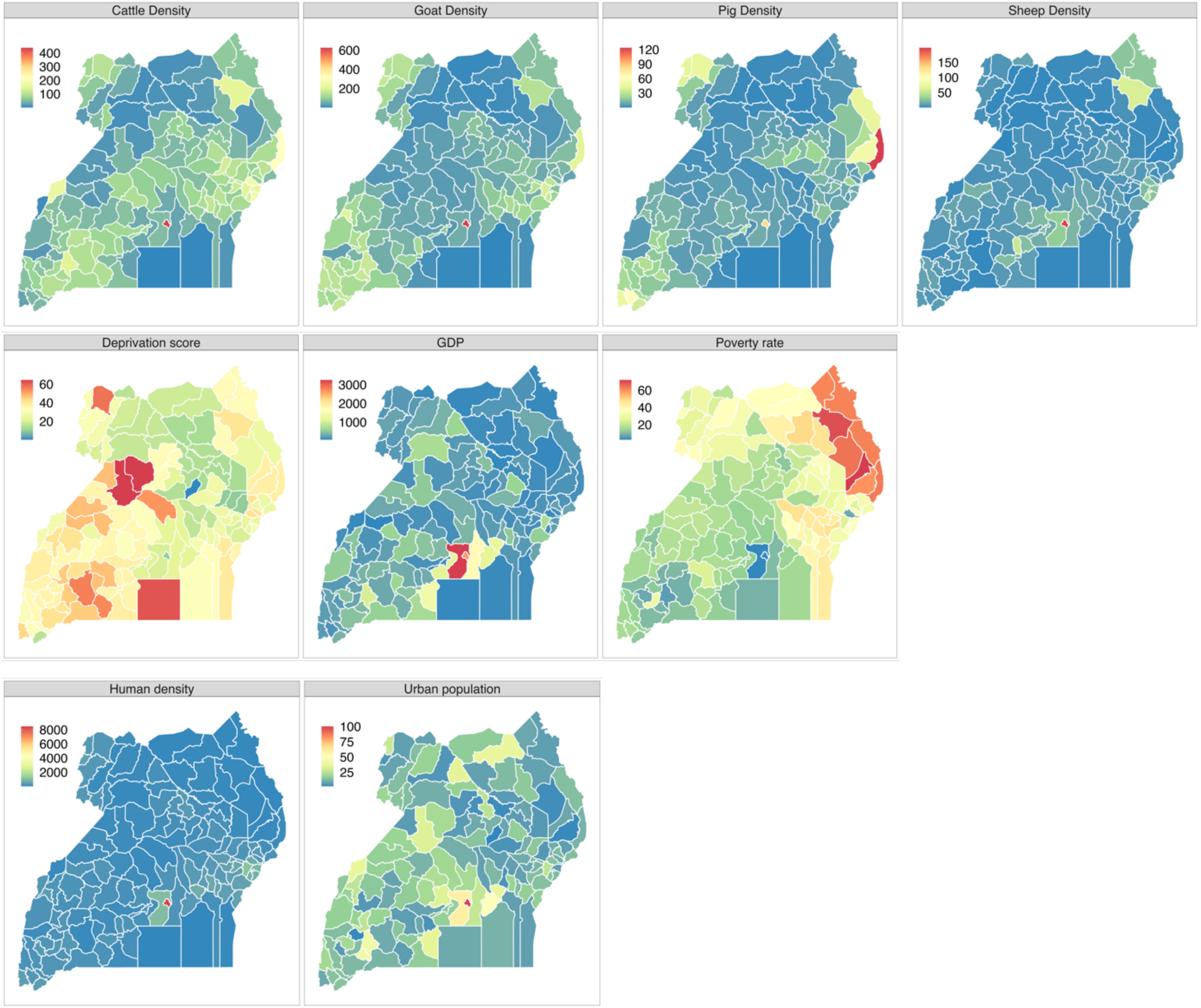

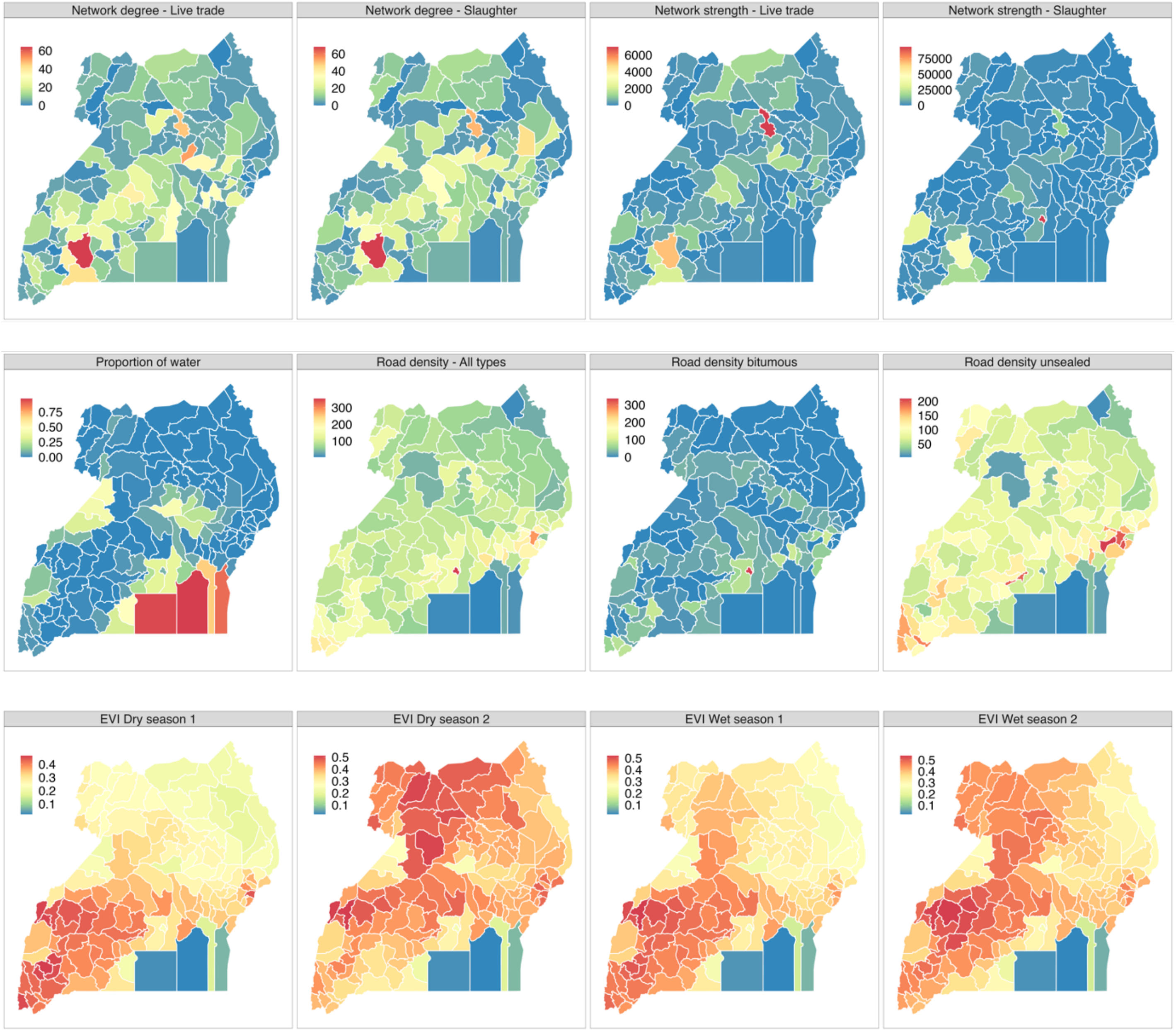
Network degree - Live trade
60
40
20
0
Network degree - Slaughter
60
40
20
0
Network strength - Live trade
6000
4000
2000
0
Network strength - Slaughter
75000
50000
25000
0
Proportion of water
0.75
0.50
0.25
0.00
Road density - All types
300
200
100
Road density bitumous
300
200
100
0
Road density unsealed
200
150
100
50
EVI Dry season 1
0.4
0.3
0.2
0.1
EVI Dry season 2
0.5
0.4
0.3
0.2
0.1
EVI Wet season 1
0.5
0.4
0.3
0.2
0.1
EVI Wet season 2
0.5
0.4
0.3
0.2
0.1

Additional file 4. Additional details of FMD Bayesian Hierarchical Model (BHM) parametrization

The background risk (Component a), or expected outbreak count (e_i), was calculated by standardizing outbreaks counts to the number of villages at risk per district:

$$\boldsymbol{R} = \frac{\sum \boldsymbol{y_i}}{\sum \boldsymbol{p_i}} \text{ and } \boldsymbol{e_i} = \boldsymbol{p_i R}$$

where p_i is the number of villages and y_i the outbreak count per district (i). The expected counts were assumed to be fixed over the period of study and represent the district-level expected number of outbreaks that would have arisen from the underlying population if it behaved like the overall standard (Elliott et al., 2001; Moraga, 2020).

The Standardized Morbidity Ratio (SMR), a crude estimate of the local RR, was defined for each district as the ratio between observed and expected outbreak counts:

$$\boldsymbol{SMR} = \frac{\boldsymbol{y_i}}{\boldsymbol{e_i}}$$

The SMR was used to map the outbreaks prior to model fitting, providing a first, spatially adjusted impression of FMD risk across the country. It can be interpreted as the 'excess' risk of outbreaks relative to expectation, where it suggests higher (SMR>1), lower (SMR<1) or equal (SMR=1) risk before accounting for other eco-epidemiological factors that may influence local disease risk (Moraga, 2020).

To better address overdispersion and to account for the possibility of unobserved heterogeneity between districts, we extended the general structure of the model by incorporating the possibility of uncorrelated (UH) and correlated (CH) spatial heterogeneity (Bernardinelli et al., 1995; Lawson, 2021). In practice, UH is the district-specific model effect that accounts for the possibility of clusters emerging within the district, in other words, uncorrelated noise at the district level. In contrast, the CH refers to the variation in risk that extends across neighbouring districts, based on a matrix built on shared geographical borders. This spatial structure was first introduced by Besag *et al* (1991) and is known as the Besag-York-Molliè (BYM) convolution model (Besag et al., 1991), commonly used for disease mapping (Madden, McGrath, et al., 2021; Schrödle et al., 2011; Schrödle & Held, 2011; Wijayanti et al., 2016). The model was extended to include a second coarser spatial effect, the subregion, due to the variation in the reported number of outbreaks. This nested regional spatial structure has been documented by Shrödle & Held (2011) and used to model Coxiellosis in Switzerland (Schrödle & Held, 2011). In this analysis, the subregion was modelled as an 'independent and identically distributed Gaussian random effect (iid)' which in practical terms means incorporating the possibility of UH occurring at the subregional level.

Additional file 5. Spatial model structures for FMD disease mapping before adding covariables. Comparison of model fitting results for different model structures**.** NB: Negative binomial; ZINB: Zero-inflated negative binomial; ZIP: Zero-inflated Poisson.

Model	DIC	WAIC	Marginal Likelihood	Dispersion statistic
Outbreaks ~ BYM (District)				
Poisson	421	422	-343.86	0.28
NB	528	528	-348.88	4.73
ZIP	485	486	-358.00	1.12
ZINB	529	529	-347.29	3.56
Outbreaks ~ BYM (District) + iid (Subregion)				
Poisson	421	422	-224.08	0.31
NB	500	499	-225.00	2.69
ZIP	463	461	-232.64	0.73
ZINB	529	529	-224.15	3.55

Additional file 6. Univariable analysis for variable selection Spatial BHM Spatial Poisson – District. Variables in which the posterior distribution did not overlap 0 with an 80% Credible Interval (CrI) were moved forward to build a global (full) model. Prior to inclusion in the global model, variables were assessed for multicollinearity, and only one form (e.g. quartiles, log-transformed) was retained for analysis.

	**Spatial BHM Spatial Poisson**		
**Parameter (Animal Demographics)**	**Posterior mean**	**80% CrI**	
Cattle density	1.5909	0.8491	2.3406
Cattle density categories			
Q5	Ref.		
Q4	0.4123	-0.0965	0.9195
Q3	-0.5972	-1.1978	0.0034
Q2	-0.3201	-0.8997	0.2578
Q1	-1.3144	-2.0159	-0.6199
Sheep density	-0.4639	-0.9235	-0.0037
Sheep density categories			
Q5	Ref.		
Q4	-0.4288	-1.0772	0.2176
Q3	-0.311	-0.987	0.3614
Q2	0.5247	-0.0993	1.1486
Q1	0.5595	-0.1064	1.2248
Goat density	0.6552	-0.0551	1.3715
Goat density categories			
Q5	Ref.		
Q4	0.6005	-0.0523	1.2558
Q3	0.6202	-0.0355	1.2788
Q2	0.8049	0.0872	1.5278
Q1	0.219	-0.5667	1.0025
Pig density	0.4892	-0.0936	1.0771
Pig density categories			
Q5	Ref.		
Q4	0.6411	-0.0846	1.3718
Q3	0.8324	0.1	1.5713
Q2	0.467	-0.2778	1.2131
Q1	0.226	-0.627	1.0792
**Parameter (Environmental)**	**Posterior mean**	**80% CrI**	
Mean of mean EVI 1st Dry season	0.2176	-3.3021	3.7651

Mean of mean EVI 1st Dry season categories			
Q5	Ref.		
Q4	0.3477	-0.3563	1.0502
Q3	0.6795	-0.2056	1.5667
Q2	0.4509	-0.5235	1.4253
Q1	-0.1766	-1.2146	0.8548
Mean of mean EVI 2nd Dry season	-0.1529	-3.1388	2.8419
Mean of mean EVI 2nd Dry season categories			
Q5	Ref.		
Q4	0.226	-0.3925	0.8451
Q3	0.8734	0.1854	1.5662
Q2	1.1201	0.4301	1.8151
Q1	0.8825	0.1597	1.6112
Mean of mean EVI 1st Wet season s	0.7995	-2.3315	3.9473
Mean of mean EVI 1st Wet season categories			
Q5	Ref.		
Q4	0.5464	-0.1153	1.2045
Q3	0.4064	-0.3936	1.2059
Q2	-0.2334	-1.1217	0.6496
Q1	0.5863	-0.2939	1.4643
Mean of mean EVI 2nd Wet season	0.609	-2.3582	3.5947
Mean of mean EVI 2nd Wet season categories			
Q5	Ref.		
Q4	0.1307	-0.4972	0.7537
Q3	0.0085	-0.6933	0.7075
Q2	0.0469	-0.6563	0.7488
Q1	0.5268	-0.2165	1.2708
**Parameter (Socio-economic)**	**Posterior mean**	**80% CrI**	
Deprivation score	0.046	0.0282	0.064
Deprivation score categories			
Q5	Ref.		
Q4	-0.3422	-0.8826	0.2007
Q3	-0.6628	-1.264	-0.0603
Q2	-0.5567	-1.1515	0.039

Q1	-2.1889	-2.9681	-1.4124
Poverty rate	0.0313	0.0097	0.0529
Poverty rate categories			
Q5	Ref.		
Q4	-0.9081	-1.6042	-0.2134
Q3	-0.1662	-0.9314	0.603
Q2	-0.2058	-1.074	0.6661
Q1	-0.0908	-1.0276	0.8505
GDP per capita	-0.1473	-0.7236	0.4293
GDP per capita categories			
Q5	Ref.		
Q4	0.6611	0.1294	1.1923
Q3	0.2536	-0.386	0.896
Q2	0.5116	-0.056	1.0809
Q1	0.0773	-0.5317	0.6852
**Parameter (Trade and commerce)**	**Posterior mean**	**80% CrI**	
Human density	-0.905	-1.5228	-0.2833
Human density categories			
Q5	Ref.		
Q4	-0.2922	-1.0512	0.467
Q3	0.3985	-0.321	1.1201
Q2	0.5112	-0.2537	1.277
Q1	0.9799	0.1778	1.7782
Urban population categories			
Q5	Ref.		
Q4	0.4003	-0.232	1.032
Q3	0.5792	-0.0305	1.1891
Q2	0.7078	0.1076	1.308
Q1	0.6671	0.0569	1.2781
Border	-0.2979	-0.7889	0.1926
Network strength categories			
Q5	Ref.		
Q4	-0.0502	-0.6083	0.5122
Q3	-0.6355	-1.2354	-0.0337
Q2	0.5795	0.0203	1.1442
Q1	-0.1081	-0.7158	0.5009
Network degree categories			
Q5	Ref.		
Q4	-0.3815	-0.9072	0.1464
Q3	0.2689	-0.2307	0.7705

Q2	-0.646	-1.2568	-0.0354
Q1	0.5136	-0.1172	1.1462
**Parameter (Spatial accessibility)**	**Posterior mean**	**80% CrI**	
Road density (all types)	-0.001	-0.0054	0.0034
Road density (all types) categories			
Q5	Ref.		
Q4	0.4738	-0.1714	1.1178
Q3	0.3752	-0.3137	1.0627
Q2	0.2672	-0.4368	0.9701
Q1	0.6697	-0.0654	1.4028
Road density (bitumous)	1e-04	-0.0067	0.0071
Road density (bitumous) categories			
Q5	Ref.		
Q4	0.311	-0.2758	0.897
Q3	-0.1388	-0.7323	0.4517
Q2	0.2422	-0.3733	0.8589
Q1	0.5855	-0.0629	1.2334
Road density (unsealed)	0.001	-0.0047	0.0068
Road density (unsealed) categories			
Q5	Ref.		
Q4	-0.6362	-1.287	0.0154
Q3	0.2489	-0.3695	0.8679
Q2	0.4899	-0.1618	1.1429
Q1	-0.2329	-0.9003	0.4334
Proportion of water categories			
Q5	Ref.		
Q4	0.6458	0.0028	1.2893
Q3	0.9885	0.2825	1.6943
Q2	0.4736	-0.2127	1.1601
Q1	0.8923	0.2184	1.5667
**Parameter (Vaccination)**	**Posterior mean**	**80% CrI**	
Mean vaccination coverage	11.012	7.211	14.849

Additional file 7. Comparison of alternative BHM Spatial Poisson – District/Subregion models. Only selected best-performing models are shown.

Model	Network centrality metric	Poverty	Deprivation	EVI Mean Dry2	Regional	WAIC	DIC	Dispersion	Marginal log-likelihood
global2	Strength	Rank	Rank	Rank	No	433.19	418.29	2.35	-280.41
global6**	Degree	Rank	Rank	Rank	No	432.57	417.66	2.37	-272.19
global6_a	Degree	Excluded	Rank	Rank	No	429.4	418.26	2.41	-265.08
global6_b	Excluded	Rank	Rank	Rank	No	429.79	418.46	2.39	-265.04
global6_reg*	Degree	Rank	Rank	Rank	Yes	431.41	417.12	2.37	-280.74
global6_reg_a*	Degree	Excluded	Rank	Rank	Yes	428.96	418.11	2.4	-265.37

*Region as a non-structured random effect (iid)

**Final model

Appendix 8. SMR comparison *BHM Spatial Poisson – District*. (Top) Map of district-level adjusted spatial standardised morbidity ratio (SMR) estimates from the best-fitting model (BHM Spatial Poisson – District). (Bottom) District-level baseline SMR and fitted values using the best model (*BHM Spatial Poisson – District).*

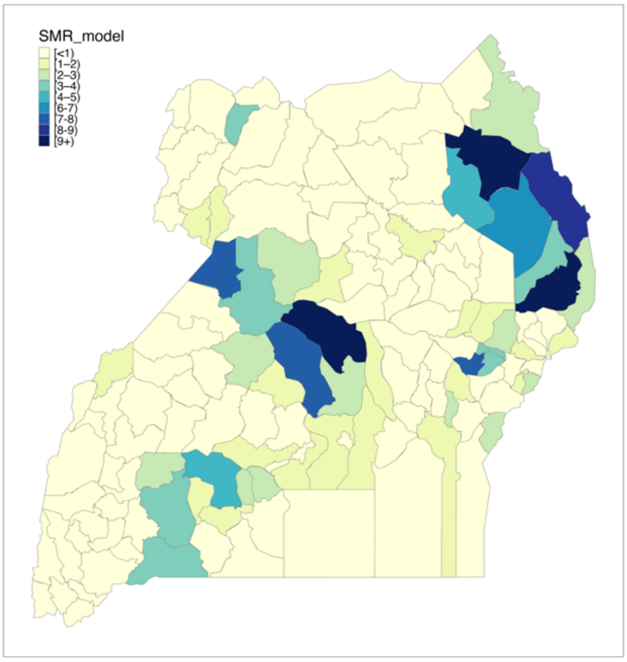

District	SMR baseline	SMR model	SMR model 95% CrI	
Abim	4.33	4.05	1.67	8.3
Adjumani	0	0.41	0.02	1.98
Agago	0	0.07	0	0.31
Alebtong	1.79	1.23	0.4	2.91
Amolatar	0	0.3	0.03	1.16
Amudat	1.48	2.81	0.55	8.67
Amuria	0	0.23	0.02	0.87
Amuru	0	0.02	0	0.12
Apac	1.52	1.15	0.41	2.59
Arua	0	0.08	0.01	0.35
Budaka	3.34	3.35	1.16	7.68
Bududa	0.93	0.92	0.32	2.12
Bugiri	0.58	0.85	0.15	2.7
Bugweri	3.33	2.08	0.35	6.96
Buhweju	0	0.07	0	0.34
Buikwe	0	0.37	0.04	1.41
Bukedea	4.13	2.77	0.6	8.14
Bukomansimbi	2.63	2.72	0.79	6.92

Bukwo	1.27	1.19	0.3	3.24
Bulambuli	0.67	0.69	0.25	1.56
Buliisa	8.52	7.86	2.91	17.27
Bundibugyo	0	0.02	0	0.14
Bunyangabu	0	0.07	0	0.44
Bushenyi	0	0.08	0	0.36
Busia	2.46	2.1	0.84	4.4
Butaleja	0.54	0.93	0.19	2.76
Butambala	0	0.4	0.01	2.16
Butebo	3.19	3.31	1.17	7.51
Buvuma	0	0.36	0.01	1.87
Buyende	0.63	0.39	0.05	1.44
Dokolo	0	0.13	0.01	0.58
Gomba	1.65	1.84	0.47	5.04
Gulu	0	0.05	0	0.28
Hoima	0.72	0.84	0.21	2.3
Ibanda	0	0.07	0	0.33
Iganga	0	0.33	0.03	1.34
Isingiro	3.97	3.43	1.94	5.63
Jinja	1.08	0.8	0.16	2.47
Kaabong	1.61	2.03	0.78	4.34
Kabale	0	0.02	0	0.14
Kabarole	0	0.08	0	0.39
Kaberamaido	0	0.18	0.01	0.83
Kagadi	0	0.11	0.01	0.5
Kakumiro	1.09	0.69	0.13	2.21
Kalaki	0	0.06	0	0.36
Kalangala	0	0.69	0.02	3.72
Kaliro	0	0.17	0.01	0.75
Kalungu	3.16	2.71	0.85	6.58
Kampala	0.26	0.36	0.04	1.36
Kamuli	0	0.08	0	0.39
Kamwenge	0	0.15	0.01	0.62
Kanungu	0	0.02	0	0.1
Kapchorwa	0.34	0.49	0.07	1.73
Kapelebyong	0.75	0.46	0.04	1.97
Karenga	0	0.42	0.01	2.18
Kasese	0	0.04	0	0.25
Kassanda	0	0.38	0.04	1.44
Katakwi	0	0.4	0.05	1.49
Kayunga	1.75	1.8	0.57	4.33

Kazo	2.93	2.59	0.86	6.09
Kibaale	0	0.13	0.01	0.65
Kiboga	0.92	1.46	0.31	4.34
Kibuku	8.14	7.12	3.41	13.18
Kikuube	0	0.44	0.05	1.71
Kiruhura	3.49	3.49	1.75	6.26
Kiryandongo	3.8	2.76	0.82	6.92
Kisoro	0	0.03	0	0.21
Kitagwenda	0	0.14	0	0.76
Kitgum	0	0.04	0	0.21
Koboko	0	0.09	0	0.58
Kole	0	0.16	0.01	0.68
Kotido	15.64	15.01	8.22	25.25
Kumi	2.65	1.66	0.29	5.41
Kwania	0	0.64	0.08	2.29
Kween	0.45	0.95	0.21	2.74
Kyankwanzi	2.29	2.34	0.86	5.19
Kyegegwa	0.46	0.24	0.02	0.99
Kyenjojo	0	0.11	0.01	0.48
Kyotera	0.59	0.7	0.19	1.84
Lamwo	0	0.05	0	0.3
Lira	0	0.11	0.01	0.49
Luuka	0	0.34	0.03	1.44
Luwero	2.62	2.49	1.11	4.85
Lwengo	1.92	1.44	0.46	3.44
Lyantonde	1.01	1.79	0.37	5.33
Madi okollo	0	0.12	0.02	0.42
Manafwa	0.93	1.05	0.46	2.05
Maracha	0	0.07	0	0.45
Masaka	0	0.57	0.07	2.08
Masindi	3.58	3.93	1.65	7.97
Mayuge	1.32	1.26	0.39	3.11
Mbale	0.47	0.53	0.13	1.45
Mbarara	0	0.35	0.06	1.1
Mitooma	0.4	0.18	0.02	0.77
Mityana	0.35	0.42	0.07	1.41
Moroto	10.27	8.99	3.89	17.86
Moyo	0.98	0.83	0.08	3.49
Mpigi	1.3	1.56	0.42	4.13
Mubende	0.83	0.69	0.24	1.57
Mukono	1.13	1.15	0.37	2.75

Nabilatuk	2.33	3.88	0.76	11.94
Nakapiripirit	18.46	17.88	10.15	29.28
Nakaseke	7.72	7.37	4.1	12.26
Nakasongola	9.66	9.2	5.23	15.05
Namayingo	0	0.58	0.04	2.46
Namisindwa	2.26	2.17	1.06	3.98
Namutumba	1.28	1.59	0.44	4.1
Napak	7.15	6.56	3.09	12.3
Nebbi	1.99	1.82	0.85	3.45
Ngora	1.62	1.56	0.2	5.86
Ntoroko	1.13	1.22	0.14	4.67
Ntungamo	0.45	0.47	0.13	1.25
Nwoya	0	0.06	0	0.32
Obongi	4.34	3.67	0.95	9.91
Omoro	0	0.03	0	0.18
Otuke	0	0.26	0.02	1.06
Oyam	0	0.26	0.04	0.87
Pader	0	0.03	0	0.14
Pakwach	1.19	1.19	0.47	2.52
Pallisa	0.38	0.87	0.23	2.25
Rakai	0.3	0.6	0.13	1.78
Rubanda	0	0.03	0	0.21
Rubirizi	0	0.07	0	0.37
Rukiga	0	0.08	0	0.47
Rukungiri	0	0.11	0.01	0.45
Rwampara	0.62	0.74	0.12	2.51
Serere	0.92	0.83	0.11	3.06
Sheema	0	0.07	0	0.34
Sironko	0.85	1.08	0.46	2.16
Soroti	0	0.37	0.03	1.52
Ssembabule	4.29	4.29	2.06	7.92
Tororo	0.56	0.65	0.16	1.81
Wakiso	1.53	1.41	0.54	3.01
Yumbe	0	0.21	0.01	0.9
Zombo	0.37	0.18	0.01	0.83

Additional file 9. Results of best model + vaccination coverage. The inclusion of mean vaccination coverage resulted in a modest improvement in model fit (DIC = 413.98; WAIC = 419.86). District-level FMD risk across the country using the best fitting vaccination model (*Model 5: BHM Spatial Poisson – District with mean vaccination coverage*) - Exceedance probabilities for the period 2014 to 2019 show similar pattern than for the same model without vaccination with a slight increase for high-risk clusters.

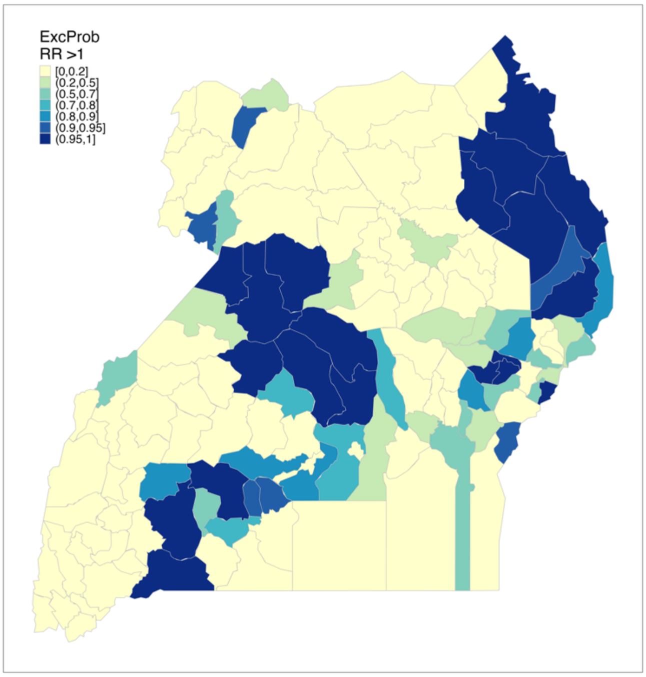

Additional file 10. Model checks. Local DIC percentile maps (top) and scatterplot (+ bar plot) of posterior means predictive distributions against observed outbreaks (bottom) for the best model (*Model 2; BHM Spatial Poisson – District)*. Lower local DIC values indicate districts where the model provides a particularly good fit. The scatterplot of the posterior means of the predictive distributions against the observed outbreak counts shows that data is reasonably well-explained by the final model (Correlation = 0.99).

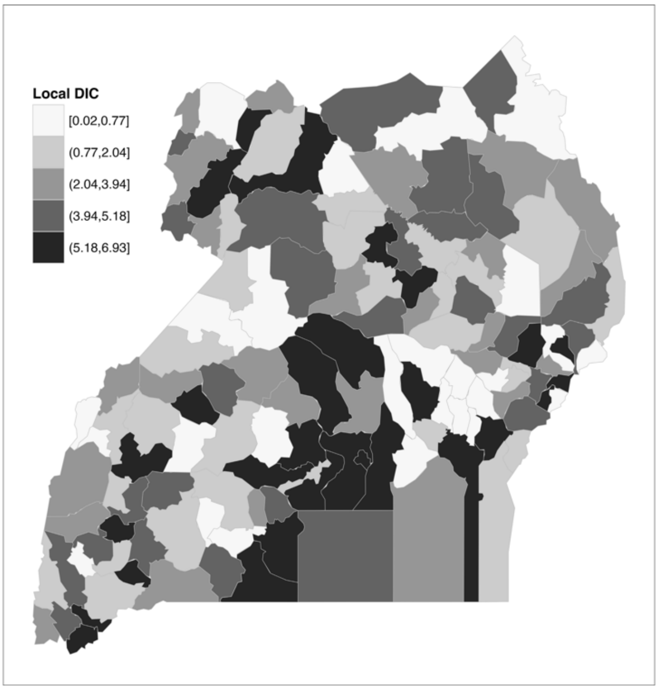

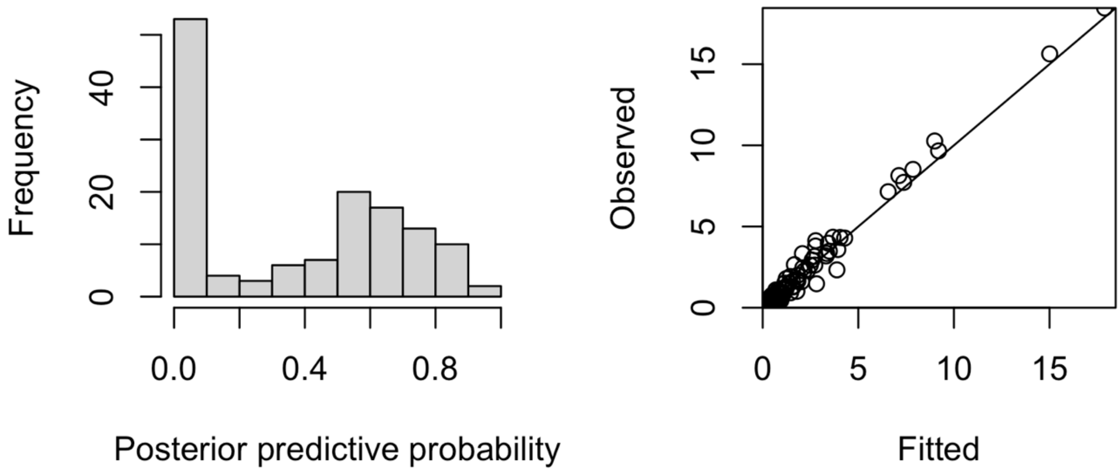

Additional file 11. Results of ZIP model. (Top) Results ZIP ecological regression models with different spatial structures. ZIP crude model showed strong evidence of residual spatial autocorrelation (Moran's I statistic, $p < 0.01$), supporting the addition of spatial random effects improved model performance. (Bottom) District-level FMD risk across the country using the best fitting ZIP model (*Model 5: BHM Spatial ZIP – District*) - Exceedance probabilities for the period 2014 to 2019 show similar pattern than for the best Poisson model (Model 2; BHM Spatial Poisson – District)

Variable	Summary: Mean ± SD	Model 1: Crude ZIP RR (95% CrI)	Model 2: BHM Spatial ZIP– District RR (95% CrI)	Model 3: BHM Spatial ZIP – District and subregion RR (95% CrI)
**Cattle density**		1.70 (0.90 – 3.19)	7.54 (2.25 – 26.84)	7.46 (2.23 – 26.31)
**Network degree category**				
Q5	40.90 ± 14.10		Ref.	
Q4	18.20 ± 3.29	0.70 (0.43 – 1.13)	0.81 (0.33 – 2.05)	0.81 (0.33 – 2.03)
Q3	9.96 ± 1.89	0.73 (0.50 – 1.06)	1.73 (0.77 – 3.97)	1.72 (0.77 – 3.90)
Q2	5.22 ± 1.01	0.32 (0.18 – 0.55)	0.73 (0.27 – 1.99)	0.73 (0.27 – 1.95)
Q1	2.30 ± 1.38	0.62 (0.41 – 0.93)	1.22 (0.43 – 3.49)	1.22 (0.43 – 3.46)
**Human density**		0.25 (0.17 – 0.37)	0.18 (0.05 – 0.64)	0.18 (0.05 – 0.64)
**EVI 2nd Dry season category**				
Q5	0.47 ± 0.01		Ref.	
Q4	0.43 ± 0.01	0.98 (0.58 – 1.65)	1.04 (0.37 – 2.89)	1.03 (0.37 – 2.86)
Q3	0.40 ± 0.01	1.03 (0.65 – 1.62)	2.18 (0.75 – 6.55)	2.18 (0.75- 6.49)
Q2	0.37 ± 0.01	0.86 (0.54 – 1.38)	2.94 (1.00 – 9.03)	2.94 (1.00 – 8.85)
Q1	0.28 ± 0.09	0.58 (0.36 – 0.93)	1.93 (0.63 – 6.23)	1.92 (0.63 – 6.17)
**Deprivation score category**				
Q5	47.62 ± 7.48		Ref.	
Q3	35.95 ± 1.22	0.50 (0.33 – 0.74)	1.07 (0.42 – 2.80)	1.07 (0.42 – 2.77)
Q3	29.48 ± 2.69	0.79 (0.52 – 1.19)	0.58 (0.22 – 1.57)	0.58 (0.22 – 1.55)
Q2	22.57 ± 2.05	0.66 (0.44 – 1.01)	0.84 (0.31 – 2.23)	0.83 (0.31 – 2.20)
Q1	13.94 ± 3.68	0.60 (0.31 – 1.15)	0.20 (0.06 – 0.71)	0.20 (0.06 – 0.71)
**Poverty category**				
Q5	49.41 ± 10.42		Ref.	
Q4	32.37 ± 2.88	0.53 (0.35 – 0.80)	0.70 (0.22 – 2.29)	0.70 (0.22 – 2.27)
Q3	20.95 ± 3.05	0.48 (0.29 – 0.79)	1.72 (0.49 – 6.17)	1.72 (0.49 – 6.05)
Q2	15.58 ± 0.90	0.50 (0.30 – 0.82)	1.30 (0.33 – 5.16)	1.28 (0.34 – 5.05)
Q1	10.31 ± 3.10	0.51 (0.32 – 0.80)	1.99 (0.44 – 9.30)	1.97 (0.44 – 9.21)
**Model fit**				
DIC		587.12	420.79	420.47

WAIC	625.26	421.56	421.18

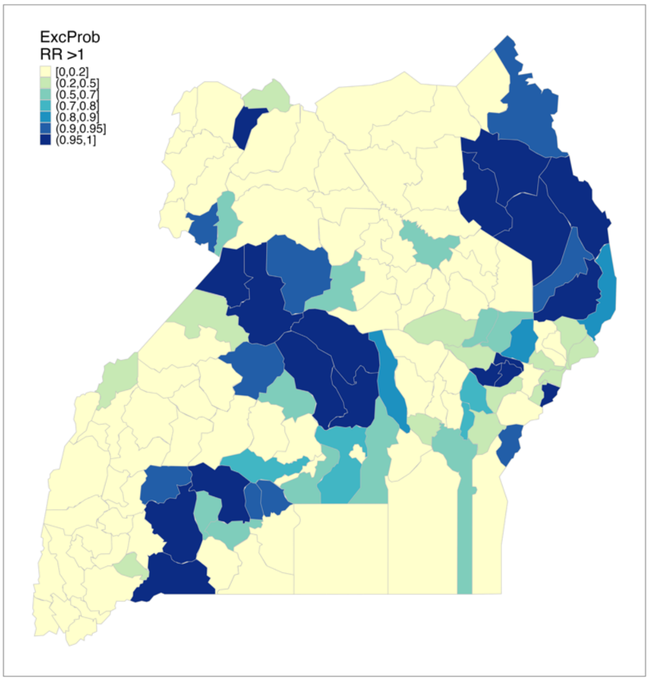

Supplement: Supplementary file 1 — Supporting Information Additional tables, figures, and supplementary material relevant to the analyses presented in this manuscript. [file TBED-2026-4994209-s001.pdf]
